# Research progress on the cross-regulation between ferroptosis and immunogenic cell death in tumor micro-environment

**DOI:** 10.3389/fonc.2025.1581951

**Published:** 2025-06-04

**Authors:** Weitao Liu, Yichen Jing, Yang Chen, Han Sun, Wenbo Xu, Ruihan Liang, Wanglin Liu, Zengyu Zhang, Huiping Liu

**Affiliations:** ^1^ Hunan Provincial Key Laboratory of Traditional Chinese Medicine Oncology, Hunan University of Traditional Chinese Medicine, Changsha, Hunan, China; ^2^ Department of Foreign Languages, University of Chinese Academy of Sciences, Beijing, China; ^3^ Research Center for Clinical Medicine, Jinshan Hospital Affiliated to Fudan University, Shanghai,, China

**Keywords:** ferroptosis, cross regulation, tumor microenvironment, immunogenic cell death (ICD), damage associated molecular pattern molecules (DAMPS)

## Abstract

Ferroptosis and immunogenic cell death, as two unique forms of cell death, have attracted extensive attention in the biomedical field. Recent studies have shown the synergistic effect of ICD and ferroptosis in the tumor microenvironment, where tumor cells undergo immunogenic cell death and release immunogenic molecules, such as DAMPs, to recruit and activate immune cells and promote adaptive immune responses. At the same time, molecules such as lipid peroxides produced by ferroptosis may also enhance the anti-tumor activity of immune cells. In addition, the synergistic use of ferroptosis and ICD in combination with novel protocols such as biomaterials and nanotechnology has demonstrated promising anti-tumor effects. This article reviews the cross-regulatory mechanism of ferroptosis and ICD in the tumor microenvironment, and explores the related biological effects between immune cells and ferroptosis, and the potential application of the two in the treatment of cancer. At the same time, we put forward insights into the solution of the existing problems in the combination of ferroptosis and ICD, as well as new ideas and development directions for future cancer treatment.

## 1 Introduction to ferroptosis

Tumor micro-environment (TME) refers to a comprehensive environment in which the occurrence, growth and metastasis of tumor are closely related to the internal and external environment of tumor cells. Tumor microenvironment affects tumor cell metabolism, immunity and therapeutic effect, and plays a core role in tumor occurrence and development (1). Although the research on tumor microenvironment has made remarkable progress, there are still some limitations. For example, the understanding of the interaction mechanism between various components in tumor microenvironment is still not deep enough, and the targeted therapy methods for tumor microenvironment still need to be explored and optimized (2). In recent years, Ferroptosis and immunogenic cell death (ICD), as two unique ways of cell death, have attracted wide attention in the biomedical field. Ferroptosis and immunotherapy have shown great potential in the field of cancer treatment (3). Inducing ferroptosis and targeting immune cells are new strategies for cancer treatment (4). Ferroptosis is characterized by the accumulation of a large number of lipid peroxide products, especially phospholipid hydroperoxide, which will destroy the integrity of cell membrane and eventually lead to cell death. And it is a unique form of cell death because its mechanism is significantly different from the traditional cell death pathway (5). It has been proved that it is closely related to the occurrence, development and metastasis of tumors, and its potential application value in anti-tumor therapy is increasingly prominent. ICD is a special mode of cell death (6). By releasing DAMPs, antigen presenting cells (such as dendritic cells, calreticulin (CRT) and heat shock protein (HSPs)) can be recruited and adaptive immune response of T cells can be activated, so as to induce tumor cell death and establish immune memory. This way of death plays an important role in the treatment of tumor cancer, because it can promote the recognition and clearance of tumor cells and enhance the anti-tumor immune response (7). Recent studies have shown that ferroptosis and ICD have a synergistic effect in tumor microenvironment. Molecules such as lipid peroxide produced by ferroptosis may also enhance the anti-tumor activity of immune cells. Ferroptosis treats tumors through iron metabolism, lipid metabolism and amino acid metabolism, and also has some effects on the function and activity of immune cells in tumor microenvironment (8). Ferroptosis can regulate ICD through T cells, B cells, neutrophils and macrophages in tumor microenvironment. ICD not only inhibits tumor cells, but also has certain influence on tumor microenvironment (9). When tumor cells experience ICD, they will release immunogenic molecules, such as DAMPs, recruit and activate immune cells, and promote adaptive immune response. CD8+ T cells in immune cells promote ferroptosis of tumor cells through interferon-γ (IFN-γ), forming positive feedback and enhancing anti-tumor effect (10). The metabolism and polarization of T cells and macrophages affect ferroptosis. The molecular mechanism of cross-regulation of ferroptosis and ICD provides a new potential target for tumor treatment. It is beneficial to the implementation of clinical combined treatment strategies such as the combination of ferroptosis inducer and ICD inducer, and provides more effective choices for the treatment of cancer. In this paper, the cross-dialogue mechanisms between ferroptosis and ICD in tumor microenvironment are systematically summarized, and the biological effects associated with ferroptosis in immune cells are deeply discussed, and the potential application prospects of these mechanisms in the field of cancer treatment are further analyzed. By targeting the molecular mechanism of ferroptosis and ICD, we can provide ideas for developing new anti-tumor drugs and treatment schemes, improve the therapeutic effect of tumors and reduce side effects.

### 1.1 The concept of ferroptosis

Ferroptosis is a unique form of iron-dependent cell death, which is caused by excessive accumulation of lipid peroxide on the cell membrane (5). Since its first discovery in 2012, it has attracted wide attention because of its unique cellular characteristics and its potential role in cancer treatment (11). Different from apoptotic cells, iron-dead cells show the characteristics of cell membrane rupture, bubbling, mitochondrial volume reduction, intima density increase, crista reduction or disappearance, adventitia rupture and so on (12). And release DAMPs (13). Its core regulatory mechanism includes the axis of solute carrier family 7 member 11(SLC7A11)- glutathione-GPX4 and the endosome sorting complex ESCRT-III which is necessary for transport (14). SLC7A11 participates in the reverse transport of cystine/glutamic acid in cells, and generates glutathione (GSH), which is an important antioxidant in cells, and reduces lipid peroxide through GPX4 to protect cells from ferroptosis (15). Inhibition of SLC7A11 or GPX4 activity will lead to GSH depletion and lipid peroxidation accumulation (16), causing ferroptosis. In the process of ferroptosis, the mechanism of endosome sorting complex endosomal sorting complex required for transport (ESCRT)-III, which is necessary for transport, participates in limiting plasma membrane damage (14).

Studies have shown that ferroptosis is closely related to iron, lipid and amino acid metabolism. Loss of iron metabolized ferroportin (FPN) and reactive oxygen species (ROS) (17). Deletion of PLs, peroxidation of PLS in lipid metabolism (18). The chemical substances arachidonic acid and adrenal acid (AdA), amino acid metabolized GPX4 inactivation and glutathione depletion are all important factors of ferroptosis. In addition, ferroptosis also affects inflammatory reaction and tumor growth *in vivo*. Many small molecular drugs such as eRAStin (SLC7A11 inhibitor) and RSL3 (GPX4 inhibitor) can specifically induce ferroptosis, especially for cancer cells carrying mutant ras oncogene (19).

Its specific mechanism remains to be further studied. Generally speaking, ferroptosis, as a unique form of cell death, has great potential in tumor treatment. In-depth study of its regulatory mechanism and its interaction with the immune system is expected to provide new ideas for cancer treatment.

#### 1.1.1 Iron metabolism

Iron is an important element for life activities and participates in many life regulation processes. Ferroptosis, that is, iron-dependent cell death, is closely related to iron metabolism disorder. *In vivo*, Free Fe^3+^ in blood forms a complex with extracellular transferrin (Tf) and binds to transferrin receptor 1(TfR1) on cell membrane (20), enters cells through endocytosis, and Fe is reduced to Fe by iron reductase (such as Six-Transmembrane Epithelial Antigen of Prostate 3 (STEAP3)) (21). After that, Fe^2+^ was transported to the cytosol by divalent metal ion transporter 1 (DMT1). In the cytosol, most Fe^2+^ is released to the outside of the cell through FPN1 or stored by ferritin. However, when FPN is missing or ferritin function is abnormal, it will cause iron metabolism imbalance, which will lead to intracellular iron overload, lead to excessive Fe^2+^ entering the unstable iron pool (LIP) in the cytosol, generate a large number of ROS through Fenton reaction, destroy lipid peroxidation, and eventually lead to ferroptosis (22).

Therefore, iron chelating agents and nitrogen oxides can inhibit Fenton reactions, such as deferoxamine (DFO) and TEMPO, thus interrupting ferroptosis (23). In addition, the mutation or abnormal expression of genes related to iron metabolism may also affect the sensitivity of ferroptosis pathway. For example, iron response element binding protein 2 (IRB2), a key regulator of iron metabolism, can participate in PUFA peroxidation, reducing the sensitivity of cells to ferroptosis, and inhibiting IRB2 can inhibit ferroptosis (24).

In addition, ferritin autophagy is a process regulated by autophagy-related (ATG) proteins, which is related to the interaction between autophagy and lysosomes, and nuclear receptor coactivator 4(NCOA4) is its special molecule. During the autophagy of ferritin, ferritin combines with NCOA4 through FTH1 subunit to form a complex. After that, the double-walled autophagy membrane contains ferritin -NCOA4 complex, forming a completely closed structure and transferring to lysosomes, where ferritin degrades and releases iron. When iron metabolism is disordered or ATG expression is abnormal, iron autophagy mediated by NCOA4 can induce ferroptosis by degrading ferritin and inducing iron overload (25).

Hypoxia can also affect iron metabolism. Hypoxia leads to the increase of erythropoietin (EPO) and serum TF, which eventually leads to abnormal iron metabolism, thus promoting ferroptosis. In addition, hypoxia can enhance the level of HIF-1 and promote the concentration of transferrin to regulate ferroptosis (23).

#### 1.1.2 Lipid metabolism

Lipid metabolism plays a central role in ferroptosis. Lipid peroxidation is a process in which ROS oxidizes biofilm after oxidative stress is enhanced, and it is a key factor driving cell death (26). There are two ways to trigger lipid peroxidation: non-enzymatic or enzymatic. Enzymes mean that lipid peroxidation can occur through lipoxygenase (LOX), cyclooxygenase (COX) and cytochrome P450 (CYP). Non-enzymatic means that lipid peroxidation can occur in a non-enzymatic way through free radical-induced peroxidation, autooxidation and photodegradation (25).

The accumulation of polyunsaturated fatty acids (PUFA) is considered as a sign of ferroptosis (27). Because PUFA is the site of oxidative lipid damage, it is a necessary substance to perform ferroptosis. There are a lot of PUFA in the cell membrane, which are esterified by acyl-CoA synthetase long chain family member 4 (ACSL4), modified by lysophosphatidylcholine acyltransferase 3 (LPCAT3) and integrated into cell membrane phospholipids. These lipids are easily attacked by free radicals. For example, PUFA in lipid membrane is highly sensitive to oxidative stress, which can react with ROS and induce lipid peroxidation to form L-ROS, while high concentration of L-ROS will trigger oxidative stress in cells and lead to oxidative damage. Lipid peroxides are extremely destructive to cells, because they will destroy the thickness, permeability and structure of membrane bilayers. Lipid peroxidation occurs and lipid hydroperoxide (PL-OOH) is generated, which leads to membrane damage and ferroptosis (25, 28, 29). Studies have shown that exogenous monounsaturated fatty acids (MUFA) can inhibit the accumulation of lipid ROS on plasma membrane and replace PUFA on plasma membrane, thus effectively inhibiting ferroptosis (30, 31). In addition, studies have shown that phosphatidylethanolamine (PE) is the key phospholipid to induce cell ferroptosis. PE is involved in biosynthesis and reconstruction by ACSL4 and lysophosphatidylcholine acyltransferase 3 (LPCAT3), which can activate PUFA and affect its transmembrane characteristics (31, 32).

At the same time, the decomposition products of lipid peroxide, including 4- hydroxynonenal (HNE) and malondialdehyde (MDA), will also damage the cell process, because they form adducts with protein and DNA, thus affecting the normal function of cells (30).

#### 1.1.3 Amino acid metabolism

The occurrence of ferroptosis is closely related to the depletion of intracellular GSH and the failure of antioxidant enzyme GPX4. System Xc-, as the reverse transport protein of cystine/glutamic acid, is very important for amino acid metabolism.

System Xc- a heterodimer composed of two subunits, SLC3A2 and SLC7A11, can transport cystine into cells and release glutamic acid out of cells. In the cell, cystine is first reduced to cysteine, and then enters the cell with the help of transporter SLC7A11 to participate in the synthesis of GSH, thus enhancing the activity of GPX4, the main antioxidant enzyme. When Xc- is inhibited, the concentration of intracellular cysteine decreases, which limits the synthesis speed of GSH, increases the level of intracellular ROS, leads to the accumulation of lipid peroxide, and finally leads to ferroptosis. Therefore, the function of system Xc- has a direct impact on GSH level and GPX4 activity (33, 34). Based on this, we can develop drugs that can inhibit or promote ferroptosis, such as cisplatin, which inhibits the activity of GPX4 (35). In addition, amino acid metabolism also involves other pathways related to ferroptosis. For example, some amino acids can regulate ferroptosis by affecting the production of ROS or the activity of antioxidant enzymes, such as cysteine as GSH raw material (36).

### 1.2 The pathway of ferroptosis

#### 1.2.1 GSH-GPX4 pathway

The fundamental cause of ferroptosis is the imbalance of redox balance between oxidant and antioxidant, which is driven by the abnormal expression and activity of various redox enzymes in the process of producing free radicals and lipid oxidation products or detoxification. When the antioxidant system can’t withstand iron overload, excessive ROS will attack sensitive fatty acids, trigger peroxidation, destroy the integrity of cell membrane, increase oxidative stress *in vivo*, and destroy DNA, protein and lipids, thus aggravating lipid metabolism disorder and further causing ferroptosis (37). At the same time, LPO produced by lipid peroxidation, such as malondialdehyde and 4- hydroxynonenal, can induce lipid peroxidation of phospholipid-containing cell membrane, and then induce ferroptosis (38, 39). GPX4 can reduce the amount of ROS, reduce phospholipid hydrogen peroxide (PL-OOH) to phospholipid hydroxylate, maintain the redox state of GSH, prevent the accumulation of lipid reactive oxygen species, and then regulate the occurrence of ferroptosis. When GPX4 activity is lost or its function is impaired, ROS accumulation leads to lipid peroxidation, which in turn leads to ferroptosis (39, 40). Therefore, regulating the levels of GPX4 and GSH can change the occurrence and development of ferroptosis. Glutathione is the most abundant reducing agent, which affects the biogenesis of iron and sulfur clusters, and is also an auxiliary factor of many enzymes (including GPX and glutathione S transferase) (41).

#### 1.2.2 NADPH-FSP1-CoQ10 pathway

NADPH-FSP1-CoQ10 pathway is another key anti-ferroptosis pathway. The N-terminal of FSP1 contains myristic acylation domain, which has the function of lipid modification, enriching it in plasma and reducing the sensitivity of cells to ferroptosis (42). Previous studies have confirmed that FSP1 is a nicotinamide-adenine dinucleotide phosphate (NADP-)-dependent coenzyme Q(CoQ) oxidoreductase, which can be used as an electron carrier and a fat-soluble antioxidant (43). Recent studies have found that FSP1 and GPX4 cooperate to inhibit ferroptosis by directly regulating the antioxidant system of non-mitochondrial coenzyme Q10 (44).

According to Doll et al.’ s research, overexpression of apoptosis-inducing factor Mitochondrial Associated 2(AIFM2, also known as FSP1) can reverse ferroptosis induced by GPX4 inhibition, which indicates that FSP1 has nothing to do with the mechanism of GPX4 (42). Therefore, the combined inhibition of FSP1 and GPX4 is expected to be an effective strategy for the treatment of ferroptosis-related diseases.

#### 1.2.3 P62-Keap1-Nrf2 signal transduction pathway

Other molecular signal pathways related to ferroptosis, including p62-Keap1-Nrf2 signal transduction pathway, bind to Kelch-like ECH-related protein 1(Keap1) under oxidative stress, and remain inactive through ubiquitination in proteasome, and then release Nrf2 from the coupled Keap1 protein and transfer to the nucleus (45). Its core lies in that the nuclear factor erythroid 2-related factor 2(Nrf2) has antioxidant function and can participate in the regulation of ferroptosis (11, 45). Sun and his team’s research confirmed that p62-Keap1-Nrf2 signal transduction pathway has antioxidant effect on ferroptosis of hepatocellular carcinoma cells, which mainly depends on the localization mechanism of p62 as autophagy receptor, and activates Nrf2 by inactivating Keap1 (46). Nrf2 prevented ferroptosis under the mediation of NQO1, family oxygenase -1 (HO-1) and ferritin heavy chain (FTH1) (5), which indicated that ferroptosis was indirectly related to autophagy. It has been reported that p53 down-regulates the expression of SLC7A11 to conduct metal-dependent apoptosis signals and affect Xc- system, thus inhibiting the ferroptosis process. It has been found that the activation of p53 will reduce the antioxidant capacity of cells, leading to ferroptosis, which can be reversed by the treatment of ferritin -1 (Fer-1) (47). It can be seen that Nrf2 regulates the antioxidant reaction and its interaction with autophagy, while p53 regulates ferroptosis by regulating SLC7A11 expression and ROS metabolism.

Ferroptosis involves many molecules and signal pathways, and these mechanisms provide a new perspective and strategy for studying cell death and treating diseases related to ferroptosis.

### 1.3 Ferroptosis reagent

#### 1.3.1 Ferroptosis Inducer

##### 1.3.1.1 Targets small molecules and drug inducers of iron metabolism and lipid metabolism

Compared with normal cells, tumor cells are more dependent on iron, so they are highly sensitive to ferroptosis. This process can also be induced by drugs that modulate lipid and iron metabolism. For example, Withaferin A and FINO2 induce ferroptosis by inhibiting GPX4 and changing iron metabolism, respectively (48, 49). In addition, Song et al. showed that temozolomide (TMZ) could induce ferroptosis in glioblastoma cells through DMT1-dependent pathway (50). In addition, t-BuOOH-induced cell death can be saved by intercellular contact through the Hippo pathway (51). Nanomaterials partially loaded with iron can also cause ferroptosis in tumor cells (52). The recently discovered inducer, MMRi62, has been shown to induce the degradation of ferritin heavy chain, thus promoting ferroptosis (50). In the treatment of hepatocellular carcinoma, sorafenib, as an FDA-certified anticancer drug, can induce ferroptosis in the presence of ACSL4 acyl-CoA synthetase long chain family member 4 (53). Especially, the intervention of sorafenib will directly affect the metabolic pathway of lipid ROS production in cells. T-BuOOH can directly affect the level of lipid ROS, leading to DNA damage, oxidative stress and mitochondrial membrane potential imbalance, and its mechanism involves the oxidation of cardiolipin. Cardiolipin oxidation inhibitors XJB-5–131 and JP4–039 can reverse this process (53).

##### 1.3.1.2 Targets small molecules and drug inducers of GSH/GPX4 axis and FSP1/CoQ related pathways

Some tumors have failed the pathway of sulfur transfer due to mutation or apparent modification, so tumor cells are highly dependent on System Xc- for cystine uptake, and System Xc- has become an important target of anti-tumor drugs (54, 55). Erastin can effectively inhibit the growth of cervical cancer and ovarian cancer cells by regulating the formation and oxidation of GSH (56). In addition, Erastin also consumes GSH and leads to degradation of GPX4 (12, 57). Sorafenib, an anti-tumor drug approved by FDA, promotes the occurrence of ferroptosis in cells by inhibiting the function of System Xc- (58). Buthionine sulfoximine (BSO) inhibits the activity of glutamyl-cysteine ligase and blocks the production of GSH, thus inhibiting the growth of breast tumors in mice (59) and enhancing the chemosensitivity of melanoma and glioma (60, 61). RSL3 is the most commonly used GPX4 inhibitor, which can effectively promote ferroptosis in fibrosarcoma and multiple cell models (56). NDP4928 is an ferroptosis enhancer (62), and its cytotoxicity is significantly improved when it is combined with RSL3 or BSO. The target of NDP4928 is FSP1, which binds to and inhibits FSP1, and defines the ferroptosis pathway induced by GSH inhibition (63). FOIN56 is a multi-effect inducer, which not only induces ferroptosis by degrading GPX4, but also induces ferroptosis by combining with squalene synthase (SQS) and consuming CoQ (64). Statins are formulated into therapeutic nanoparticles (65), which can inhibit the synthesis of CoQ10 by blocking HMGCR in mevalonate pathway and trigger ferroptosis after reducing CoQ (66). As shown in **Table 1**.

**Table 1:** Ferroptosis inducer form.

Medicine	Mechanism	Experimental model	Related genes	Cite
Withaferin	Inhibit GPX4 activity, degrade heme and increase iron content.	Human neuroblastoma cells IMR-32 and SK-N-SH	CPX4	Song Q et al. (50)
FINO2	Inhibiting GPX4 and ferric oxide	Human fibrosarcoma cell HT-1080, human immortalized skin tissue cell BJeLR.	CPX4	Faust D et al. (51)
Temozolomide (TMZ)	Decreased the expression of Nrf2 and HO-1, and up-regulated DMT1.	Human glioblastoma cell line TG905	DMT1, SQLE, GPX4	Song Q et al. (50)
Iron-based nanomaterials	Inhibition of tumor growth by Fenton reaction	MMTV-PyMT (mouse breast tumor virus-T antigen in polyoma) breast cancer cells	TfR1、FPN1、HO-1	Chen M et al. (54), Geppert M et al. (239)
Sorafenib	Inhibition system Xc enhances intracellular iron content.	Hepatocellular carcinoma (HCC) tissue	SLC711, STAT3,MCL1	Dhiman P et al. (55)
Erastin	Inhibition of System Xc-, interference with cystine uptake, decrease of GSH and increase of lipid peroxidation level.	Human cervical cancer cell Hela, human ovarian cancer cell COV362	p53	Xiong Y et al. (59), Wu H et al. (60)
Buthionine sulfoxide imine (BSO)	Inhibit the activity of glutamyl-cysteine ligase and block the production of GSH.	Neuroblastoma cell line	p53,GPX4,ATM	Cunningham A et al. (244), Yoshioka et al. (63), Chen X et al. (64)
RSL3	Cysteine binding to GPX4 active site inhibits GPX4 activity.	Human fibrosarcoma cell HT-1080 and nude mice transplanted tumor model	GPX4,SLC711,SLC3A2	Tang R et al. (52)
Statins	Inhibit GPX4	Triple negative breast cancer (TNBC) cells	GPX4	MILLÁN M et al. (68)
FIN56	Inhibit GPX4;; Combined with squalene synthase (SQS), CoQ is consumed.	Four kinds of engineered BJ cell lines, 143B cells (osteosarcoma), Calu-1 cells (lung adenocarcinoma) and HT-1080 cells (fibrosarcoma).	SLC7A11 GPX4 FSP1	Deepa S et al. (67), Costa I et al. (245)

#### 1.3.2 Ferroptosis inhibitor

##### 1.3.2.1 Small-molecule inhibitors that reduce the ferroptosis

Iron chelating agents, such as DFO and dexrazoxane (DXZ), can selectively target intracellular Fe^2+^ and prevent ROS produced by Fenton reaction, thus achieving the effect of inhibiting ferroptosis (67, 68). DXZ is approved by FDA for DIC treatment of cancer patients, which can relieve heart dysfunction by inhibiting ferroptosis. In addition, there are other iron ion chelating agents, such as DFA1, BMS536924 and purine analog 2, which show higher efficiency and lower side effects in inhibiting ferroptosis. At the same time, there are ferroptosis inhibitors that directly reduce the level of Fe^2+^, such as YL 939, which can reduce the iron level in intracellular LIP by regulating ferritin expression and ferritin phagocytosis, thus inhibiting ferroptosis. According to the research of YANG W et al. (69), this compound can effectively inhibit cell death and inflammatory infiltration in acetaminophen-induced liver injury model. Hirata et al. (70) synthesized a class of compounds containing N,N- dimethylaniline structure, targeting secondary endosomes and lysosomes, and inhibiting ferroptosis by reducing Fe^2+^. Among them, GIF-2–114 and GIF-2 197-r), the two compounds with the highest activity, have the same neuroprotective ability as Fer-1 on cell death induced by glutamate and Erastin at sub-nano molar concentration. Ferristatin II can promote the degradation of TfR1, interfere with Tf-mediated iron delivery, and has also been proved to inhibit ferroptosis (71), which provides a new strategy for the treatment of various nervous system diseases. Fang et al. (72) found a kind of ferroptosis inhibitor 9a targeting NCOA4. By binding with NCOA4, it destroyed the protein-protein interaction between NCOA4 and FTH1, blocked iron autophagy dependent on NCO A4, and reduced Fe^2+^ in intracellular LIP, thus inhibiting ferroptosis.

##### 1.3.2.2 As a small molecular inhibitor for reducing lipid peroxide

The first ferroptosis inhibitor, Fer 1, was found by Qualcomm screening, which can quickly react with peroxy free radicals in cells and block the amplification of chain reaction in lipid peroxidation, thus inhibiting ferroptosis (73). Lip-1 is a spiroquinoxaline amine derivative with the same mechanism as Fer-1. Because of its good absorption and distribution, ferroptosis can be effectively inhibited at a lower dose (74). α -tocopherol (α-TOH) is the most bioactive form of vitamin E, and it is also an effective ferroptosis inhibitor. The reactivity of its analogue THNs is 100 times higher than that of Fer 1 and Lip-1 in organic solution, but its ferroptosis inhibitory activity in cells is similar to that of them (75).

##### 1.3.2.3 Small-molecule inhibitors affecting the GSH/GPX 4 axis

β-mercaptoethanol (m-ME) has an ferroptosis inhibitory effect in System Xc-it can prevent the cell from being blocked in the process of cystine uptake (34). In this process, β-ME reacts with cystine to generate mixed disulfide, which is transmitted to cells through system L, and cystine is rapidly generated, thus accelerating the generation of GPX4 (76). Increasing the expression of GPX4 can effectively resist ferroptosis, which is regulated by selenium (Se) and can be achieved by activating transcription factors TFAP2c (transcription factor activating protein 2γ) and Sp1 (specific protein 1). However, TFAP2c may be affected by transcription inhibitors, and cells with low GPX4 level cannot be protected by Se (77). Partner-mediated autophagy can induce the degradation of GPX4, but triterpenoid 2- amino -5- chloro -N, 3- dimethylbenzamide (CDDO) can inhibit the degradation of GPX4 by inhibiting molecular chaperone HSP90, thus preventing cells from ferroptosis (78). A new diphenyl butene inhibitor compound 3f discovered by Fang et al. (79) can inhibit ferroptosis by increasing the level of FSP1 protein. In addition, many natural antioxidants, such as 43 kinds of curcumin, 26 kinds of baicalin, 44 kinds of resveratrol and 45 kinds of sulforaphane (80), have been proved to have the activity of inhibiting ferroptosis because of their polyphenol structure. As shown in **Table 2**.

**Table 2:** Ferroptosis inhibitor form.

Medicine	Mechanism	Experimental model	Related genes	Cite
Iron chelating agent	It can selectively target Fe2+ in cells and prevent ROS produced by Fenton reaction.	Middle cerebral artery occlusion (MCAO) model in rats	GPX4, ACSL4,	Kosyakovsky J et al. (246), MILLÁN M et al. (68)
YL 939	Regulate ferritin expression and ferritin phagocytosis, and reduce the iron level in intracellular LIP.	Acetaminophen-induced liver injury model	PHB2	Wang Y et al. (74)
Ferristatin II	Promote the degradation of TfR1 and interfere with Tf-mediated iron delivery.	Mouse TBI model	TfR1, 、 Tf	Wang Y et al. (74)
9a	Destroy the interaction between NCOA4-FTH1 protein and protein to reduce the amount of bioavailable intracellular ferrous iron.	Rat model of ischemic stroke	NCOA4-FTH1	Fang et al. (72)
Fer-1	Quickly react with peroxy free radicals in cells to block the amplification of chain reaction in lipid peroxidation.	Cell models of Huntington’s disease (HD), periventricular leukomalacia (PVL) and renal dysfunction	SLC711,GPX4	Li Z et al. (247)
Lip-1	ROS produced by lipid peroxidation	Mouse melanoma cells	EGRI,SLC711,GPX4	Bao C et al. (248)
β-MErcaptoethanol (β-me)	Increase GPX4 expression.	Mouse lymphoma L1210 cells	Transcription factors TFAP2c, HSP90,	Fang Y et al. (79), Gu Y et al. (80)
Novel diphenyl butene inhibitor compound 3f	Increase FSP1 protein level	Rat model of ischemic stroke	FSP1	Fang et al. (79)

## 2 Introduction of immunogenic cell death

### 2.1 The concept of an immunogenic cell death

Immunogenic cell death is a regulatory programmed cell death pattern specially designed to activate the adaptive immune response of syngeneic hosts with sound immune function (81). The function of ICD is to trigger adaptive immune response, trigger stress response of organelles and cells, and finally lead to apoptosis (82). Molecules released by dying or stressed cells, such as ATP, HMGB1, CRT and proinflammatory cytokines, can all be used as adjuvants or danger signals of the immune system, and such signals are collectively called DAMPs (83). The release of DAMPs is a remarkable feature of ICD. When DAMPs are exposed to the extracellular membrane or released to the extracellular matrix in a specific time and space, it can combine with its corresponding pattern recognition receptors (PRR), such as TLR, NLRs, RLRs, etc., so as to deliver immune stimulation signals, stimulate downstream signal transduction pathways, start cell cascade reactions, and attract antigen-presenting cells. For example, when TLR4 recognizes HMGB1, it will activate MyD88 signal pathway. RIG-I-like receptor can activate interferon regulatory factor IRF 3/7 after recognizing viral RNA; NOD-like receptors promote the release of IL-1β and IL-18 by forming inflammatory corpuscles (84), further stimulate the proliferation and activation of T lymphocytes, greatly enhance their anti-tumor benefits, and finally trigger innate and adaptive immune responses, resulting in strong and lasting anti-cancer immunity (85–89).

#### 2.1.1 ICD antigenicity of ICD

In the process of ICD, antigenicity plays a key role, which determines whether the dead cells can be effectively recognized by the host immune system and the efficiency of triggering immune response. Antigenicity mainly comes from four aspects: 1. Bacterial and viral infection: Infection by pathogens (such as viruses and bacteria) can lead to the production of a large number of antigenic determinants. Microbial proteins are not covered by central tolerance, and their epitopes are highly antigenic, which can bind to host T cell receptor (TCR) and initiate immune response (90, 91). 2. Tumor cell mutation: Most human tumors are not driven by active virus infection, but malignant cells are often accompanied by an increase in gene mutation rate during immune escape (92, 93). These mutations include non-sense point mutation, insertion deletion and so on, which lead to the exposure of new tumor antigen (TNA). TNA is quite different from its own epitope structure, and some of them are similar to microbial epitopes, which can effectively trigger ab initio immune response. In addition, cancer cells also express some tumor-associated antigens (TAA), such as CD19, CD20, PMEL and MLANA (94, 95). These antigens also exist in healthy tissues, but due to incomplete central tolerance, they can still trigger immune response under the background of strong adjuvant. 3. Post-translational modification (PTM): antigenic determinants can also be produced by PTM, such as phosphorylation, acetylation, glycosylation, etc (96). These modifications can change the structure of protein and produce new epitopes, which may not be covered by central tolerance, so they can trigger immune response under certain conditions. 4. Endogenous retroviruses: Normal cell genomes contain a large number of endogenous retroviruses, which are usually dormant under physiological conditions. However, under stress conditions, these viruses may be activated and express antigen proteins, giving healthy cells certain ICD capabilities (97, 98). ICD process is an immune response triggered by cell death. The key is that DAMPs released after cell death can stimulate immune cells such as antigen presenting cells (APC) and T cells, leading to programmed cell death. Therefore, if we can inhibit the activation or function of immune cells, or block the release pathway of DAMPs (such as HMGB1 and ATP), it is expected to weaken the immune response caused by ICD. For example, by inhibiting the three key genes LY96, BCL2 and IFNGR1 in SAP and using antagonists of immune checkpoint inhibitors, it may be helpful to control the activation of T cells, thus reducing the impact of immune response on ICD (99). However, due to the variety of DAMPs and the complex mechanism of action, the research on inhibitors of DAMPs release is still in the primary stage. It is expected that with the in-depth understanding of the release mechanism of DAMPs, such inhibitors are expected to become new therapeutic strategies.

In addition, cytokines involved in ICD (such as IFN-γ and IL-1β) and death receptors on the cell surface (such as FAS and TNFR) can also be used as key factors affecting the immune effect (100, 101).

#### 2.1.2 ICD adjuvant nature of ICD

Adjuvant is another key aspect in ICD process, which determines whether dying cells can effectively recruit and APC, and then start adaptive immune response. Adjuvant properties are mainly provided by DAMPs, including ATP, HMGB1, CRT, HSPs and so on (82). These molecules are released or exposed to the cell surface during cell death. These molecules are released or exposed during cell death and are recognized by the immune system as “danger signals”.

##### 2.1.2.1 DAMPs mechanism of DAMPS

###### 2.1.2.1.1 ATP

ATP, as a cell energy carrier, is released to the outside of the cell during ICD, and serves as a “find-me” signal to recruit DC and macrophages to the tumor area to promote the infiltration of immune cells. Its release mechanism includes autophagy dependence and ERS induction. ATP-loaded vesicles are released in a autophagy-dependent manner through Pannexin channels. In ERS state, ATP release may be related to the activation of PERK/eIF2α signaling pathway (102). The main function of ATP is to recruit and activate immune cells. By binding to DC and P2RY2 receptor on the surface of macrophages, ATP promotes the infiltration of many myeloid cells into tumor areas (103). In addition, it can activate NLRP3 inflammatory corpuscles in DC, promote the secretion of IL-1β and IL-18, and activate CD8+ T cells and γδT cells (104).

###### 2.1.2.1.2 HMGB1

As a kind of DAMP, HMGB1 has immune stimulation. HMGB1 released from the nucleus to the extracellular matrix binds to TLR4 on the surface of DC, which enhances the processing and cross-presentation of antigen by DC and activates the specific T cell immune response (84). The release mechanism includes active release and passive release. Active release is the exocytosis pathway mediated by specific lysosomal vesicles, or active release in the process of cell death mediated by caspase-1/caspase-11; Passive release is the late stage of apoptosis, and the permeability of cell membrane increases, which leads to the passive release of HMGB1 to the extracellular space (105).

###### 2.1.2.1.3 CRT

As an “eat-me” signal, CRT promotes the phagocytosis of apoptotic cells by macrophages. CRT is a Ca-binding protein resident in endoplasmic reticulum (ER), which is involved in protein folding, ER homeostasis and MHC class I molecule assembly (106). During ICD, CRT is transferred from ER to the surface of cell membrane, which promotes the phagocytosis of apoptotic cells by immune cells. CRT has the functions of phagocytosis signal and antigen presentation, and interacts with CD91 and TLR4 on DC surface to drive macrophages to phagocytize apoptotic cells (82, 107). The release mechanism includes ERS induction and vesicle transport. ERS-induced translocation of CRT is caused by ER stress (ERS) activating unfolded protein reaction (UPR). Under the interaction of SNARE and SNAP23/25, CRT translocates to cell membrane through exocytosis (108); As for vesicle transport, CRT binds to vesicle or plasma membrane-related proteins, and transports from ER to Golgi apparatus in the form of vesicles, and then transports to cell membrane (109).

###### 2.1.2.1.4 HSPs

HSPs not only protect cells from damage, but also combine with damaged protein to form immune response complex and activate immune cells. HSPs are molecular chaperones activated under stress, including HSP60, HSP70, HSP90, etc., which play a key role in the survival of cells in environmental stress injury. In the process of ICD, HSPs will be translocated to the cell membrane or passively released to the outside of the cell as immunostimulating molecules. HSPs has two main functions. First of all, HSPs can be used as an immune adjuvant, combined with damaged protein, to enhance the recognition and presentation of antigens by DC (110). Secondly, HSPs can promote phagocytosis, and HSPs on cell membrane, as an “eat-me” signal, promote phagocytosis of apoptotic cells by immune cells. The release mechanism of HSPs includes ERS induction and passive release. In ERS state, HSPs may participate in the process of apoptosis through Fas/caspase8 pathway and be exposed on the cell membrane (111), while HSPs may be passively released into extracellular matrix during cell death.

### 2.2 Immunogenic cell death pathway

#### 2.2.1 ROS-based endoplasmic reticulum stress-related pathway activation

ERS, as a comprehensive stress response, is closely related to ICD. Under ERS condition, unfolded protein reaction (UPR) is activated, which mainly activates protein kinase R-like endoplasmic reticulum kinase (PERK) through glucose regulatory protein 78(GRP78)/binding protein (BiP). PERK further phosphorylates eukaryotic translation initiation factor 2α(eIF2α), thus promoting the translation of activated transcription factor 4(ATF4) and inducing the expression of CCAAT enhancer binding protein homologous protein (CHOP). This process will then activate the expression of apoptotic proteins such as BAX, PUMA and BAK downstream, and complete the cell stress response program (112). However, some drugs such as 4- phenylbutyric acid (4-PBA) can inhibit endoplasmic reticulum stress response, which may inhibit ICD induced by ERS (113).

Overexpression of ERS and ROS is considered to be a common feature of ICD, and ICD inducers that can induce strong ERS are classified as type II inducers. Compared with other inducers, type ii inducer has stronger inducing effect, and wogonin is one of them, which can induce ERS by inducing ROS, and then induce ICD. In this process, PRK/PEKR/eIF2α, as the upstream signal of PI3K/AKT activation, can induce the release of DAMPs and activate DCs to participate in the generation of ICD (114). In addition, cardiac glycoside ICD inducers such as oleanolide can also trigger ERS by blocking Na^+^/Ca^2+^ exchange, which will enrich and promote ROS production in mitochondria, thus activating the PERK/eIF2α/ATF4/CHOP pathway and inducing ICD (115). According to the current reports, ERS-related PEKR/eIF2α/ATF4 pathway based on ROS may be the most critical upstream pathway for ICD induction.

#### 2.2.2 Inducing ICD-related pathway activation based on regulated cell death

Cell death is the basis of ICD generation. At this time, RCD-related pathways play the role of repeaters in the process of ICD generation. At present, the research on the death pathway of ICD mainly focuses on the classical pathway of apoptosis. For example, brucine can inhibit autophagy by regulating ERK/mTOR/p70S6K pathway, and further trigger ICD (116). PI3K/AKT acts as the downstream response pathway of eIF2α in the process of inducing ICD by wogonin (114). Resveratrol promotes apoptosis and autophagy by inhibiting NGFR/AMPK/mTOR pathway, thus inducing ICD (117). Cinobufagin induces ICD through the upstream STAT pathway (118). Apoptosis is the most common death form of ICD. However, in recent years, new RCD forms such as autophagy, scorching death, ferroptosis and necrotizing apoptosis have also been confirmed in ICD research. The same drug can even trigger ICD through multiple forms of death. For example, shikonin, as a proteasome inhibitor, can activate receptor and mitochondrial mediated apoptosis pathway by targeting granzyme A (GzmsA), thus triggering ICD. In addition, shikonin can mediate ferritin phagocytosis by regulating aspartate aminotransferase (GOT), and then induce ICD (119, 120). Necrotic apoptosis is considered to be a highly immunogenic way of death, and enhanced autophagy is usually accompanied by necrosis. It is found that shikonin can stimulate RIPK1 and RIPK3-dependent necrosis and directly promote the up-regulation of DAMPs through autophagy, in which ROS accumulation and NF-κB pathway activation may be the main mechanisms of cell necrotizing apoptosis (121). Coke death is due to the perforation of gasdermins (GSDMs), which destroys the continuity of cell membrane. This death mode is more conducive to the release of DAMPs such as HMGB1 than apoptosis, and has a natural pro-inflammatory advantage. When ferroptosis occurs, ferrous ions will start liposome peroxidation through Fenton reaction, which will lead to the accumulation of ROS in cells, thus it is easier to induce strong ERS. The discovery of immunogenic ferroptosis broadens the concept of immunogenic cell death at present and opens up new possibilities for cancer treatment.

### 2.3 Immunogenic cell death drugs

#### 2.3.1 ICD application of ICD activator

##### 2.3.1.1 Chemotherapy and radiotherapy

The main form of ICD is apoptosis, but recently, new RCD methods such as autophagy, coke death, ferroptosis and necrotizing apoptosis have gradually emerged. The reason is that such drugs as adriamycin and daunorubicin can not only directly inhibit the synthesis of DNA and RNA, kill tumor cells, but also induce ICD in tumor cells. In this process, tumor cells will release DAMPs (such as HMGB1, ATP, CRT, etc.), which can be used as signals of “eat me” and “find me” to attract and activate DCs and other immune cells, thus triggering adaptive immune response.

Oxaliplatin, another platinum drug, induces tumor cell apoptosis by forming DNA adducts and triggers ICD. Similarly, the ICD induced by oxaliplatin is accompanied by the release of DAMPs, which further enhances the anti-tumor immune response.

Other chemotherapeutic drugs, such as cyclophosphamide and paclitaxel, can induce ICD to some extent and improve the anti-tumor effect, although their mechanisms of action are different. Radiotherapy directly damages the DNA of tumor cells through high-energy rays, leading to cell death. Radiotherapy can not only kill tumor cells directly, but also induce ICD and release DAMPs, thus activating the immune system. At present, the combined application of radiotherapy and immunotherapy has become a research hotspot, aiming at enhancing the effect of immunotherapy through ICD induced by radiotherapy.

##### 2.3.1.2 Immunocheckpoint inhibitors for immunotherapy drugs

Although PD-1/PD-L1 inhibitor cannot directly activate ICD function, it can effectively alleviate immunosuppression and improve the body’s ability to recognize and remove antigens caused by ICD (122). With the assistance of chemotherapy or radiotherapy, these inhibitors can significantly enhance the anti-tumor effect. In addition, tumor vaccine is also a good choice. This vaccine is based on tumor-specific antigen and can stimulate human specific immune response. When vaccine-induced T cells encounter tumor cells that trigger ICD, their ability to recognize and eliminate these cells will be greatly improved.

##### 2.3.1.3 Emerging treatment technologies

Photodynamic therapy (PDT): PDT is a non-invasive treatment method, which uses photosensitizer and light with specific wavelength to destroy tumor cells. Photosensitizers accumulate in tumor cells and are activated by light with a specific wavelength, and then generate singlet oxygen and other ROS, which destroy the membrane structure, protein and DNA of tumor cells and cause cell death. The production of reactive oxygen species can also trigger endoplasmic reticulum stress. Endoplasmic reticulum stress can lead to tumor cell apoptosis or other forms of programmed cell death. In the process of cell death, tumor cells will release DAMPs, such as CRT and HMGB1. These DAMPs can be recognized by peripheral immune cells and activate anti-tumor immune response. Therefore, PDT not only directly destroys the tumor cells in the irradiated area, but also activates the immune system of the body, recognizes and attacks distant tumor cells that are not directly affected by PDT, and achieves systemic anti-tumor immune effect. This treatment method shows great potential in the field of cancer treatment because of its advantages of high efficiency, strong selectivity and stimulating immune system.

CAR-T cell therapy: CAR-T cell therapy is a kind of cell therapy. Through genetic engineering technology, T cells of patients are transformed to express chimeric antigen receptor (CAR) which can recognize the surface antigen of tumor cells, so as to accurately target and destroy cancer cells (123). CAR-T cell therapy can not only directly kill tumor cells, but also indirectly activate the immune system by releasing cytokines. OV, as a new treatment method, can induce ICD (124–126). OV has the ability to cooperate with CAR-T cells to help them overcome many obstacles in solid tumors. Firstly, OV can release dangerous signals through ICD, reverse tumor immunosuppression, and make CAR-T cells expand, activate and recruit in TME (127). Secondly, the selective directional lysis function of OV on tumor cells leads to the lysis of infected tumor cells and the subsequent release of TAA, which triggers tumor-specific immune response and prevents tumor from escaping due to antigen deletion or antigen heterogeneity. Finally, therapeutic transgene can be inserted into OV, which is expected to enhance the effector ability of T cells (128).

#### 2.3.2 ICD application of ICD inhibitors

In some autoimmune diseases or organ transplant rejection, immunomodulatory drugs can be used to suppress excessive immune response, including ICD-induced immune response. By reducing or eliminating the stressors that can induce ICD, such as chemotherapy drugs, radiotherapy and virus infection, the incidence of ICD can be reduced. For example, using drugs to change the tumor microenvironment leads to the loss of immunosuppression and immune monitoring, which leads to the disorder of ICD (129). Hypoxia is a key factor in tumor microenvironment, which promotes immunosuppression through various mechanisms. For example, the anaerobic respiration of cells increases under anoxic conditions, which leads to the accumulation of lactic acid and forms an acidic environment, which is not conducive to the function of cytotoxic T lymphocytes (CTL) and reduces the toxicity of natural killer cells (NK cells) (130, 131). In addition, hypoxia also promotes the formation of tumor neovascularization, enhances the invasion of tumor cells, and damages the function of T cells in many ways, such as glucose deprivation, extracellular adenosine accumulation and enhanced expression of immune checkpoints (130, 132). Finally, hypoxia can also promote the survival of tumor cells by inhibiting the expression of apoptotic genes and supporting autophagy (129). Therefore, drugs aimed at hypoxic tumor microenvironment can be used to improve immune environment. However, the application of these drugs requires strict control of indications and contraindications to avoid unnecessary damage to patients. As shown in **Table 3**.

**Table 3:** Application related forms of ICD.

Genes/axes/ compounds/drugs	Mechanism	Related genes/pathways	Cite
Wogonin	Induce ROS to trigger ERS, and then induce ICD.	PRK/PEKR/eIF2α	Xie Y et al. (11)
Oleanolide	Blocking Na+/Ca2+ exchange can trigger ERS, promote the production of ROS, and then induce ICD.	PERK/eIF2α/ATF4/CHOP	Tang Z-Y, et al. (249)
resveratrol	Promote apoptosis and autophagy, thus inducing ICD.	NGFR/AMPK/mTOR	Farras M et al. (76)
Cinobufagin	Inducing ICD	Upstream STAT pathway	Liang D et al. (12)
alkannin	Activate receptor and mitochondrial mediated apoptosis pathway, thus triggering ICD.	Regulating aspartate aminotransferase (GOT) to mediate ferritin phagocytosis; Stimulate RIPK1 and RIPK3-dependent necrosis and up-regulate DAMPs; ROS accumulation, NF-κB pathway activation	Tang D et al. (14) Zhang W et al. (15)
Adriamycin, daunorubicin	Directly inhibit the synthesis of DNA and RNA, kill tumor cells, and induce ICD in tumor cells.	Tumor cells release DAMPs	Lei G et al. (5)
Oxaliplatin	The formation of DNA adducts induces apoptosis of tumor cells and triggers ICD.	Tumor cells release DAMPs	Xie Y et al. (11) Tang Z-Y et al. (249)
Chemotherapeutic drugs such as cyclophosphamide and paclitaxel.	Inducing ICD can improve the anti-tumor effect and promote the apoptosis of tumor cells.	DAMPs, PGE₂, STAT3	Zhao Y et al. (28)
PD-1/PD-L1 inhibitor	Relieve immunosuppression and improve the body’s ability to recognize and remove antigens produced by ICD.	Suppression immune checkpoint	Farras M et al. (76)
Drugs for relieving tumor hypoxia microenvironment	Improve the tumor hypoxia microenvironment and promote the normal running of immune cell function	The anaerobic respiration of tumor cells increases and lactic acid accumulates, which damages the function of immune cells; Tumor neovascularization enhances the invasion of tumor cells	Song L et al. (250)
4- phenylbutyric acid (4-PBA)	Inhibition of endoplasmic reticulum stress response, thereby inhibiting ICD induced by ERS	ROS-based endoplasmic reticulum stress-related pathway	Rellmann Y et al. (251)
adenosine	Inhibit the function of various anti-tumor immune cells and enhance the activity of immunosuppressed cells.	Adenosine -A2AR	Bao W D et al. (17), Song L et al. (250)

The purpose of using bold numerical values: References for ICD-related mechanisms and pathways.

## 3 Cross-regulation

### 3.1 Ferroptosis regulates immunogenic cell death

#### 3.1.1 Immunogenicity of ferroptosis

##### 3.1.1.1 Difference and experimental evidence of early and late ferroptosis

The immunogenicity of ferroptosis in the process of cell death shows obvious stage dependence, mainly because the release stage of DAMPs in the process of cell ferroptosis will affect the immunogenicity of cancer cells. In the immunogenicity of ferroptosis, the release of DAMPs such as ATP and HGMB1 is very important. Because of the different DAMPs content in different stages of ferroptosis in cancer cells, the immunogenicity is different. Early ferroptosis cells released a large number of DAMPs such as ATP and HMGB1, These molecules act as immune-activating “alarm signals” that activate the NF-κB signaling pathway by binding to purine-sensitive receptors (such as P2X7) and pattern recognition receptors (such as TLR4) on the surface of dendritic cells (DCs), which significantly stimulate the proliferation, activation and immune effect of DCs, thus triggering an efficient adaptive immune response (133). However, due to the depletion of key DAMPs, among which the extracellular enzymes CD39/CD73 degrade ATP to adenosine, which inhibits T cell function through the A2A receptor, and immunosuppressive factors such as TGF-β accumulate, the immunogenicity of late iron-dead cells is weakened, and it is difficult to effectively activate the immune response (134).

Efimova (135) et al. found that the cell proliferation and activation induced by ferroptosis was time-dependent with immune effect, and was closely related to the release of ATP and HMGB1 in tumor cells. They interfered with the function of purinergic receptors by oxidizing ATP (oxiATP), the 2’, 3’- dialdehyde derivative of ATP, and successfully regulated the functions of DAMPs such as extracellular ATP by inhibiting P2X7 (136–138). Related experiments have proved that in MCA205 cells at the early stage of ferroptosis, if these purinergic receptors are blocked after being induced by RSL3 for a period of time, the ability of cells to resist re-attack will be obviously weakened (138–140). These findings emphasize the important role of ATP in the immune response triggered by early iron-dead cancer cells, and the remarkable immunogenicity potential of early iron-dead cells. In addition, photodynamic therapy has also been proved to further promote the release of DAMPs and enhance the anti-tumor immune response by inducing ferroptosis.

In general, the sequential release of DAMPs during ferroptosis constitutes the molecular basis of its immunogenicity. The explosive release of DAMPs in the early stage provides the necessary starting signal for activating antitumor immunity, while the depletion of DAMPs in the late stage leads to a decline in immunogenicity. This finding provides an important theoretical basis for the development of tumor immunotherapy strategies based on the regulation of ferroptosis timing, especially in terms of optimizing the ICD process to enhance antitumor immune responses.

##### 3.1.1.2 Promoting effect of characteristic substances of ferroptosis (lipid peroxides) on ICD

Lipid peroxidation products can lead to various types of RCD, such as ferroptosis, apoptosis, and immunogenic cell death. Lipid peroxides not only act as passive byproducts but also serve as active mediators that regulate cell death pathways and influence disease pathogenesis. The promotional role of lipid peroxides in ICD has opened new avenues for targeted therapies against tumors, which can regulate lipid peroxidation to prevent or treat diseases characterized by abnormal cell death (141).

The accumulation of lipid peroxides leads to oxidative damage of cell membranes, disrupting their structural and functional integrity, and serves as an important initiating step in promoting ICD. In this process, lipid peroxides attack unsaturated fatty acids in cell membranes, generating lipid free radicals and peroxides, which trigger a chain reaction causing widespread damage to cell membranes. This increases cell membrane permeability, leading to abnormal exchange of substances between the intracellular and extracellular environments, and leakage of cellular contents such as ATP and HMGB1—DAMPs—into the extracellular environment. This triggers an immunogenic response, activates the immune system, and promotes the occurrence of ICD (142). Additionally, lipid peroxidation reactions produce large amounts of ROS. ROS not only further exacerbate lipid peroxidation reactions but also cause oxidative damage to other macromolecules within cells, such as proteins and DNA, leading to cellular dysfunction and cell death (142). Increased ROS levels can also activate multiple signaling pathways associated with ICD, such as the NF-κB pathway. Activation of NF-κB promotes the expression of pro-inflammatory genes, releases pro-inflammatory cytokines, further enhances immunogenicity, and drives the progression of ICD (143). For example, 4-hydroxy-2-nonenal (4-HNE), a product of lipid peroxidation, can upregulate Fas expression, activate caspase-3,This then triggers apoptosis, a form of the ICD (144).

Lipid peroxides can also promote mitochondrial membrane permeability by oxidatively modifying key molecules on the mitochondrial membrane, such as cardiolipin, thereby releasing cytochrome C and activating downstream apoptotic signaling pathways (145). Lipid peroxides can regulate the expression of immune-related molecules, such as by modulating the expression of thioredoxin-interacting protein (Txnip) to influence the redox state within cells, thereby regulating the survival and function of immune cells (146). In Th17 cells, the accumulation of lipid peroxides can upregulate Txnip expression, inhibit the activity of the antioxidant protein thioredoxin (Trx), further increase ROS levels, form a positive feedback loop, and accelerate Th17 cell death, a process that may be related to ICD (147, 148). In addition, the accumulation of lipid peroxides in lysosomes leads to oxidative damage to the lysosomal membrane, increasing lysosomal membrane permeability (LMP) and causing lysosomal hydrolases such as cathepsin B to be released into the cytoplasm. These hydrolases can degrade various proteins and organelles within cells, disrupting normal cell function and triggering ICD (149).

#### 3.1.2 Regulation of ferroptosis on immunogenic cells

##### 3.1.2.1 T cells

Ferroptosis, a form of iron-dependent lipid peroxidation-driven programmed cell death, plays a dual role in T cell anti-tumor immunity. The survival, expansion, and effector function of CD8+ T cells, a core effector cell population in anti-tumor immunity, are precisely regulated by GPX4. GPX4 uses reduced GSH to reduce toxic lipid peroxides (such as phosphatidylethanolamine hydroperoxide, PE-OOH) to non-toxic lipid alcohols through the selenocysteine residue in its active center, thereby maintaining cell membrane stability and blocking the ferroptosis process (150, 151). This catalytic process involves two key steps: First, the selenolate group (-SeH) of GPX4 reacts with PE-OOH to form a selenoic acid intermediate (-SeOH), while reducing PE-OOH to PE-OH; then, two molecules of GSH provide electrons to reduce the selenoic acid intermediate to -SeH, which ultimately produces oxidized glutathione (GSSG) and water (15). In follicular helper T cells (Tfh), GPX4 deficiency leads to the destruction of mitochondrial cristae structure, accumulation of lipid ROS, and subsequent ferroptosis, which ultimately weakens the germinal center response and antibody affinity maturation. It is noteworthy that selenium, an essential component of the active center of GPX4, can upregulate GPX4 expression through selenite or selenomethionine (Se-Met). Yao (150) et al. confirmed that Se-Met supplementation can increase the survival rate of GPX4-deficient Tfh cells by about 35%, and increase the antibody titer of influenza-vaccinated mice by 2–3 times, revealing the protective effect of the “selenium-GPX4 axis” on T cell function.

There is significant heterogeneity in the effects of ferroptosis on T cell subsets: CD8+ T cells are highly sensitive to GPX4 inhibitors (such as RSL3), and low doses of the drug can induce ferroptosis; however, regulatory T cells (Tregs) rely on GPX4 to resist oxidative stress after activation. Cheng et al. used the Foxp3YFP-Cre Gpx4FL/FL mouse model and found that GPX4-specific deletion in Tregs leads to lipid peroxide accumulation and triggers ferroptosis, accompanied by an increase in the proportion of Th17 cells and an enhanced anti-tumor immune response (151). This difference is due to significant differences in their metabolic characteristics: Tregs generate NADPH through the fatty acid oxidation (FAO) pathway (dependent on malate dehydrogenase and IDH1), maintaining a high GSH/GSSG ratio to resist lipid peroxidation. In addition, Tregs highly express glutaminase (GLS), which provides active support for GPX4 through glutamine metabolism. In contrast, CD8+ T cells rely on glycolysis for energy, and their rapid proliferation leads to an increase in the proportion of polyunsaturated fatty acids (such as arachidonic acid and adrenic acid) in membrane phospholipids. In addition, the insufficient amount of NADPH produced by glycolysis makes it difficult to effectively neutralize lipid peroxidation damage (152), which makes them significantly more sensitive to GPX4 inhibitors than Tregs.

In addition to GPX4, multiple molecules are involved in regulating T cell ferroptosis. The CD36 receptor, which is highly expressed on the surface of CD8+ T cells, promotes lipid droplet formation and increases the content of PUFAs in membrane phospholipids by mediating the uptake of palmitic acid and arachidonic acid, ultimately inducing ferroptosis (122). Targeting CD36 or supplementing with GSH precursors (such as N-acetylcysteine) can significantly increase the survival rate of CD8+ T cells, making them potential protective strategies. ACSL4 promotes the integration of PUFA into membrane phospholipids by catalyzing the esterification of PUFA to generate PUFA-CoA, making CD8+ T cells 2–3 times more sensitive to GPX4 inhibitors (152). It is worth noting that the immunomodulatory effect of ferroptosis is not limited to T cells themselves. For example, OTUD1 in colorectal cancer cells promotes iron uptake mediated by TfR1 by stabilizing iron response element binding protein 2 (IREB2), which exacerbates ROS production and induces ferroptosis. Metabolites such as HMGB1 and glutamate released by dead cells can reshape the immune microenvironment through a dual mechanism (153): HMGB1 binds to TLR4 on the surface of DCs, activates the NF-κB pathway to promote DC maturation and MHC molecule expression, and then activates tumor antigen-specific CD8+ T cells; glutamate indirectly promotes DC migration by regulating metabolic balance, and directly recruits tumor-reactive T cells as a chemokine factor, ultimately shifting the ratio of effector T cells/regulatory T cells in the tumor microenvironment towards the pro-inflammatory direction.

Additionally, under the stimulation of DAMPs, T cell responses exhibit a dual-track regulatory mechanism. First, DAMPs (such as HMGB1 and ATP) exert their effects through indirect activation of PRR signaling networks. Specifically, DAMPs stimulate TLR4 and NLRP3 inflammasome receptors on the surface of antigen-presenting cells such as dendritic cells, triggering upregulation of co-stimulatory molecules CD80/CD86 and secretion of cytokines such as interleukin-12 (IL-12) and interleukin-6 (IL-6), thereby creating a pro-inflammatory microenvironment that enhances T cell activation. A classic example is HMGB1 inducing Th1 cell differentiation through the TLR4 pathway, thereby enhancing antitumor immune effects (Tang et al., 2010) (154). Second, some DAMPs (such as HSP70) can directly act on the TLR2/4 receptors on the surface of T cells, regulating T cell function through a dual mechanism—promoting the proliferation of effector T cells while inhibiting the immunosuppressive activity of regulatory T cells (Tregs). This direct regulatory mechanism has demonstrated unique advantages in tumor immunotherapy (Wang et al., 2016) (155). This multi-level interaction pattern reveals the complex network of DAMPs in the regulation of immune homeostasis.

However, ferroptosis has significant bidirectional effects on immune system regulation: on the one hand, inducing Tregs ferroptosis can relieve immunosuppression and significantly enhance effector T cell activity; on the other hand, excessive activation of CD8+ T cell ferroptosis can lead to a decline in their antitumor function. This contradiction is particularly prominent in the clinical application of GPX4 inhibitors: although these drugs can enhance the immune response by clearing Tregs, their toxic effects on CD8+ T cells limit their efficacy (151, 152, 156). Key issues that need to be urgently addressed include: how to use the metabolic differences between T cell subsets (e.g., Tregs rely on FAO and CD8+ T cells rely on glycolysis) to design selective regulation strategies; the safety and efficacy of selenium supplements or CD36 inhibitors in tumor immunotherapy still need to be verified in large-scale clinical trials. Although studies have explored the therapeutic regimen of selenium compounds combined with PD-1 antibodies, the molecular mechanism still needs to be further analyzed, which provides an important research direction for the development of precise ferroptosis regulation strategies.

##### 3.1.2.2 B cells

Ferroptosis, an iron-dependent lipid peroxidation-driven form of programmed cell death, exhibits unique subset-specific effects in B cell immunoregulation. The functional heterogeneity of B lymphocytes is derived from their subset-specific biological characteristics: B cells can be divided into two major subsets, B1 and B2, based on differences in CD5 expression, and B2 cells are further subdivided into marginal zone (MZ) B cells and follicular (Fo) B cells (157). B1 cells participate in the innate immune response by rapidly secreting natural antibodies, but lack the ability to form memory cells. MZ B cells are located in the marginal zone of the spleen and are responsible for capturing circulating antigens in the blood to initiate an early immune response. Fo B cells reside in lymphoid follicles and mediate long-term immune memory by participating in germinal center responses. It is noteworthy that these subsets have significant differences in their sensitivity to oxidative stress: B1 and MZ B cells have high expression of key enzymes involved in lipid synthesis, such as acetyl-coenzyme A carboxylase (ACC) and fatty acid synthase (FASN), resulting in elevated levels of ROS in the cells, and are therefore highly dependent on GPX4 to maintain redox homeostasis (158). GPX4 binds to reduced GSH through its selenocysteine residue and catalyzes the reduction of lipid peroxides, converting the peroxyl bond into a non-toxic hydroxyl radical, thereby maintaining the stability of cell membrane lipids (159). In the absence of GPX4, the chain reaction of lipid peroxidation gets out of control, and free iron ions generate hydroxyl radicals through the Fenton reaction, leading to the destruction of cell membrane integrity. Relevant studies have shown that in GPX4 knockout models of B1 and MZ B cells, malondialdehyde (MDA) levels were significantly elevated, accompanied by increased cell membrane permeability, ultimately leading to increased cell mortality and impaired antibody secretion (160). In contrast, Fo B cells showed greater tolerance to GPX4 deficiency due to the antioxidant defense mechanism that depends on the thioredoxin system (consisting of Trx, TrxR, and NADPH). This system uses TrxR to reduce Trx with NADPH, which maintains the reduced state of intracellular proteins through disulfide bond exchange, thereby maintaining cell survival in the event of a GPX4 functional defect (161). This difference in antioxidant strategies between subpopulations provides an important molecular target for the targeted regulation of B cell immune responses.

In pathological microenvironments, the differences in ferroptosis susceptibility of B cell subsets significantly affect the anti-tumor immune response. In ovarian cancer, for example, oxidative stress in the tumor microenvironment leads to the accumulation of lipid peroxides (such as 4-hydroxy nonenal, HNE) by depleting GSH and activating 5-LOX, selectively inducing ferroptosis of B1 and MZ B cells (160). In addition, relevant studies have shown that the expression level of GPX4 in B1/MZ B cells in tumor tissue from liver cancer patients is significantly lower than that in normal tissue, while the expression of 5-LOX is significantly higher (162). Mechanistic studies have shown that oxidative stress further upregulates 5-LOX expression by activating the Nrf2 signaling pathway, forming a vicious cycle of “oxidative stress-lipid peroxidation”, which leads to impaired humoral immune function and the expansion of myeloid-derived suppressor cells (MDSCs), thereby promoting tumor immune escape (160).

The regulatory effect of ferroptosis in B-cell lymphoma shows significant subtype specificity. The germinal center B-cell-like (GCB) subtype of diffuse large B-cell lymphoma (DLBCL) is highly sensitive to the ferroptosis inducer dimethyl fumarate (DMF) due to low GPX4 expression and high 5-LOX expression (163). DMF inhibits GPX4 activity by reducing the GSH/GSSG ratio and forms a positive feedback loop with 5-LOX, which synergistically increases lipid peroxidation levels by 3–5 times and significantly induces tumor cell death. In contrast, the activated B-cell-like (ABC) subtype is resistant to ferroptosis, but DMF can enhance the killing effect of chemotherapeutic drugs by inhibiting the NF-κB and STAT3 signaling pathways and significantly downregulating the expression of the anti-apoptotic protein BCL-2. Gene expression profiling analysis shows that the expression of GPX4 and genes related to GSH synthesis in the GCB subtype is significantly lower than that in the ABC subtype, while the expression of BCL-2 in the ABC subtype is significantly higher. This molecular characteristic provides a basis for the development of subtype-specific treatment strategies (164, 165).

Under the stimulation of DAMPs, B cell responses exhibit a bidirectional characteristic of both effector and regulatory functions. On the one hand, B cells enhance humoral immune responses through pattern recognition mechanisms. For example, endogenous DAMPs (such as DNA fragments and uric acid crystals) can directly activate TLR9 receptors and NLRP3 inflammasome signaling pathways in B cells (166), driving B cells to differentiate into plasma cells and significantly enhancing the secretion of antibodies such as IgG and IgM. Specifically, mitochondrial DNA (mtDNA) as a DAMP can induce autoantibody production through the TLR9 pathway (Caielli et al., 2016) (167), revealing the triggering role of endogenous molecules in autoimmune responses. On the other hand, B cells also exhibit immune regulatory functions, responding specifically to protein-based DAMPs such as S100A8/A9 and secreting anti-inflammatory cytokines such as IL-10 to form a negative feedback regulatory loop, effectively suppressing excessive inflammatory responses (Shen et al., 2014) (168). This dual response pattern reflects the multidimensional role of B cells in DAMPs-mediated immune homeostasis regulation.

Although ferroptosis shows great potential in B cell immunomodulation and tumor therapy, the current treatment of DLBCL still faces significant challenges: the sensitivity of the GCB subtype to ferroptosis and the resistance of the ABC subtype create a therapeutic dilemma. This difference stems from the essential differences in metabolic characteristics and signaling pathways: the redox imbalance of the GCB subtype makes it vulnerable to ferroptosis, while the high expression of BCL-2 in the ABC subtype gives it an anti-apoptotic survival advantage. Solving this dilemma requires the development of precision targeting strategies, such as nanomedicine delivery systems based on surface markers of B cell subsets (such as CD10 and MUM1), which can achieve selective drug delivery by targeting specific surface antigens on tumor-associated B cells, thereby reducing damage to normal B cell subsets (169). In addition, the combination of ferroptosis inducers and BCL-2 inhibitors may be an effective strategy for overcoming subtype differences. By simultaneously targeting the ferroptosis pathway and anti-apoptotic mechanisms, the killing effect on both GCB and ABC subtypes is synergistically enhanced, thereby improving the overall treatment effect of DLBCL.

##### 3.1.2.3 Neutrophile

The core mechanism of ferroptosis is uncontrolled lipid peroxidation, and the key regulatory hub is the GSH metabolic system, in which GPX4 plays a vital role. GPX4 catalyzes the reduction of lipid peroxides through selenocysteine (Sec) in its active center, as follows: LOOH+2GSH→GPX4LOH+GSSG+H2O LOOH+2GSHGPX4LOH+GSSG+H2O

The selenol group (-SeH) of selenocysteine is directly involved in electron transfer and is 100 times more catalytically efficient than regular cysteine (15). In PMN-MDSCs (polymorphonuclear myeloid-derived suppressor cells), downregulated GPX4 expression and dysfunction of System Xc⁻ (cystine/glutamate antiporter) lead to GSH depletion, which triggers a chain reaction of lipid peroxidation (170). For example, RSL3 inhibits the activity of GPX4 by covalently binding to its Sec residue (171), while Erastin blocks System Xc⁻, further exacerbating GSH depletion. Lipid peroxidation products such as oxidized phosphatidylethanolamine (PEox) generate hydroxyl radicals through the Fenton reaction, which directly damage cell membrane integrity and release toxic aldehydes (such as malondialdehyde, MDA) (172).

Immunosuppression is mediated by multiple mechanisms, including peroxidation of PUFAs to generate PEox and prostaglandin E2 (PGE2). PEox activates Toll-like receptor 4 (TLR4), induces NF-κB-dependent PD-L1 expression, and directly inhibits CD8+ T cell function (173). PGE2 promotes the secretion of IL-10 and TGF-β by PMN-MDSCs and inhibits T cell proliferation by binding to EP2/EP4 receptors (174). Fatty acid transport protein 2 (FATP2) promotes arachidonic acid (AA) uptake and enhances the supply of lipid peroxidation substrates (175). In a FATP2 knockout mouse model, AA uptake by PMN-MDSCs was significantly reduced, resulting in decreased PEox levels and partial restoration of T cell activity (175).

The tumor microenvironment (TME) enhances the ferroptosis sensitivity of PMN-MDSCs through multiple pathways. Hypoxia stabilizes HIF-1α, which binds to the hypoxia response element (HRE) of the GPX4 promoter to inhibit GPX4 transcription (176). Tumor cells competitively uptake cystine through high expression of SLC7A1, which leads to impaired GSH synthesis in PMN-MDSCs (177). At the same time, IL-6 and TNF-α secreted by TAMs (tumor-associated macrophages) promote ROS accumulation through NOX2 activation, forming a vicious cycle of oxidative stress-ferroptosis (178). Iron-dead PMN-MDSCs highly express CD71 (transferrin receptor 1) on their surface, which transmits inhibitory signals through interactions with T cells and releases TGF-β and oxidized lipids, synergistically suppressing anti-tumor immunity (179).

Under the stimulation of DAMPs, the response of neutrophils exhibits dual characteristics of rapid response and effect amplification: on the one hand, DAMPs (such as IL-1α and ATP) activate endothelial cells, promoting the release of chemokines such as CXCL8, forming concentration gradients to guide neutrophils to migrate directionally to the injury site. Simultaneously, they directly activate the intracellular NADPH oxidase system, triggering a burst of ROS, thereby executing pathogen clearance functions (McDonald et al., 2010) (180); on the other hand, specific DAMPs (such as mitochondrial DNA and HMGB1) can stimulate neutrophils to initiate programmed cell death mechanisms, releasing extracellular traps (NETs) composed of chromatin and granular proteins. Although these reticular structures can effectively capture and inactivate pathogens, excessive activation may lead to damage to surrounding tissues (Jorch et al., 2017) (181), demonstrating the precise regulation of neutrophil function by DAMPs in acute inflammatory responses.

However, ferroptosis regulation also presents contradictions and challenges in treatment. The ferroptosis inhibitor liproxstatin-1 can reduce the immunosuppression of PMN-MDSCs, but protect tumor cells from ferroptosis, leading to an increased risk of metastasis (170). The GSH synthesis enhancer N-acetylcysteine (NAC) can restore the GSH level of PMN-MDSCs, but high doses may induce oxidative stress (182). Current solutions include targeted delivery systems, such as CD66b antibody-conjugated nanoparticles for the selective delivery of RSL3 to PMN-MDSCs; and metabolic-specific interventions, such as inhibiting CPT1A (a key enzyme in fatty acid oxidation) in PMN-MDSCs to block NADPH regeneration and enhance ferroptosis sensitivity (170). In terms of clinical application, the GPX4 agonist selenomethionine (Se-Met) upregulates GPX4 expression in a liver cancer model, and in combination with a PD-1 antibody, it significantly improves the complete response rate (CR) (183).

In summary, ferroptosis constructs an immunosuppressive network in PMN-MDSCs through the GSH depletion-GPX4 inactivation-oxidative lipid accumulation axis, providing a theoretical basis for the development of “ferroptosis-immunometabolism” dual-targeted therapy.

##### 3.1.2.4 Macrophage

The core mechanism of ferroptosis involves the dual effects of “danger signal” release and metabolic reprogramming, which regulate the remodeling of the macrophage inflammatory and polarized phenotype to reshape the tumor immune microenvironment. Lipid mediators (such as SAPE-OOH) and DAMPs such as HMGB1 and ATP released from ferroptotic cells can be used as “find me” and “eat me” specific signals to activate the macrophage immune response through pattern recognition receptors (PRRs) (184). HMGB1 activates the MyD88/TRIF-dependent NF-κB pathway by binding to the RAGE and TLR2/4 receptors, which promotes the synthesis and release of core inflammatory mediators such as IL-6 and TNF-α by macrophages (185): IL-6 activates and maintains the inflammatory response, promotes T cell proliferation and differentiation, and enhances the immune response; TNF-α has antitumor activity, induces apoptosis of tumor cells, promotes infiltration of inflammatory cells, and enhances the killing of tumor cells by immune cells (186). KRAS mutant protein and HMGB1 synergistically induce macrophage polarization to M1 type through the STING pathway, enhancing antitumor immunity (187); while SAPE-OOH directly binds to TLR2 on the surface of macrophages, enhancing the phagocytic clearance of ferroptotic cells (188). In addition, ferroptosis can induce the expression of various inflammation-related genes such as CCL2 and CCL7, thereby promoting the recruitment and chemotaxis of macrophages.

It is worth noting that the immunomodulatory effect of DAMPs is bidirectional: in hepatocellular carcinoma, HMGB1 promotes CD8+ T cell infiltration through the STING pathway, and its high expression is positively correlated with patient survival (189); however, in acute inflammation models, excessive release of HMGB1 can activate caspase-1 through the AIM2 inflammasome, promote the maturation and release of IL-1β and IL-18, and exacerbate tissue damage (190). Iron-killed tumor cells may release specific proteins (such as those encoded by the K-RasG12D gene) that are mediated by RAGE, which polarizes macrophages to the M2 phenotype through the signal transducer and activator of transcription 3 (STAT3) fatty acid oxidation pathway (187); at the same time, under certain conditions (intervention of ferroptosis inducers or KRAS mutation), 8-hydroxyguanine (8-OHG) released from iron-killed cells induces macrophages to secrete pro-inflammatory factors such as IL-6 by activating the STING pathway, promoting the infiltration of TAMs and M2 polarization (187), and forming an inflammatory environment that is conducive to tumor growth.

In the tumor microenvironment, ferroptosis drives macrophage phenotype conversion through metabolic reprogramming. KRAS mutant proteins activate mTORC1 through the RAF-MEK-ERK pathway, promote glycolysis (upregulation of HK2 and LDHA) and DNA methylation (mediated by DNMT1), and induce M2 polarization (187, 191). 8-hydroxyguanine (8-OHG) activates the cGAS-STING-IRF3 axis, which significantly increases IFN-β secretion and promotes M1 polarization. This polarization regulation is dynamically affected by microenvironmental factors: hypoxia inhibits GPX4 transcription through HIF-1α, which enhances ferroptosis sensitivity (192); tumor cells with high SLC7A11 expression block macrophage glutathione synthesis by competitively consuming cystine (177); TAMs secrete IL-6/TNF-α to activate NOX2, which exacerbates ROS accumulation and forms a positive feedback loop of “oxidative stress-ferroptosis” (187).

Additionally, macrophages exhibit synergistic characteristics of dynamic phenotypic conversion and functional remodeling in response to DAMPs: during the acute injury phase, DAMPs (such as high-mobility group box protein B1 and adenosine triphosphate) activate the Toll-like receptor 4/P2X7 receptor pathway, which in turn activates the NF-κB and NLRP3 inflammasome, driving macrophages toward M1 polarization and the secretion of pro-inflammatory factors such as interleukin-1β(IL-1β) and tumor necrosis factor-α (TNF-α), thereby establishing an efficient pathogen clearance mechanism (Gong et al., 2020) (86). In the chronic injury microenvironment, DAMPs such as fibrinogen induce the expression of arginase 1 (Arg1) and IL-10 through the integrin signaling pathway, promoting the conversion of macrophages toward the M2 phenotype to support tissue repair (Wynn et al., 2013) (193). Furthermore, DAMPs such as calretinin significantly enhance macrophage phagocytic activity through “eat me” signals such as CD91, accelerating the clearance of apoptotic cells to maintain tissue homeostasis (Gardai et al., 2005) (194).This multimodal response mechanism enables macrophages to precisely regulate between immune defense and tissue repair based on the type and duration of DAMPs.

The new delivery system shows clinical potential against the double-edged sword effect of ferroptosis therapy. Sorafenib nanoparticles wrapped in platelet membranes can specifically target TAMs, resulting in a significant decrease in the M2/M1 ratio in a pancreatic cancer model (187, 195). Exosomes modified with an antibody against CD206 deliver dimethyl fumarate (DMF) to M2 macrophages, reducing the incidence of hepatotoxicity (196). The combination of low-dose RSL3 and a PD-1 antibody significantly increased the complete response rate in melanoma (197) Notably, ferroptosis-induced immunoregulation involves epigenetic regulation: inhibition of apolipoprotein C1 (APOC1) can downregulate GPX4 activity (198), promote HMGB1 release through the MCP-UFM1-PIR axis, activate the HMGB1-RAGE-NF-κB signaling cascade (199), effectively trigger immunogenic ferroptosis, ultimately induce macrophage polarization towards the M1 type and enhance antigen presentation capacity, and produce a synergistic anti-tumor effect with PD-1 antibodies.

Current research focuses on precisely regulating the temporal and spatial effects of ferroptosis: selectively protecting normal tissue macrophages by selenomethionine to enhance the sensitivity of TAMs to ferroptosis; developing ferritin/iron transporter regulators to intervene in the balance of M1-M2 polarization; and using magnetic nanoparticles to target and induce the repolarization of M2 to M1 phenotype. These strategies aim to break through the core contradiction of ferroptosis therapy—how to induce immunosuppressive M2 macrophage ferroptosis while avoiding tissue damage caused by excessive activation of pro-inflammatory M1 type—and provide a new paradigm for solid tumor immunotherapy.

### 3.2 Immune cell death regulation ferroptosis

#### 3.2.1 Influence of immune microenvironment on ferroptosis

Immune microenvironment (TME) plays a key role in the development of tumor, and it can regulate the ferroptosis of tumor cells in many ways. Since lipid peroxidation is one of the signs of ferroptosis and is highly correlated with lipid metabolism, lipid metabolism is an important medium for ferroptosis. Intracellular fatty acids (FA) mainly come from blood and lymphatic vessels, and the level of FA is precisely regulated by the cellular conditions and external stimuli. FA oxidation is an important energy source for cancer cells. TME can affect the utilization of lipid by cancer cells through the interaction with adjacent substrates. According to this concept, lipid metabolism can regulate tumor hypoxia environment by activating hypoxia inducible factor (HIF) (200). However, in the process of tumor development, tumor cells may release some FA into TME, thus affecting the function of infiltrating immune cells, such as the transition from TAM to M2 phenotype. In addition, when lipids accumulate in DC, the antigen presenting function of DC is impaired, which further leads to the impairment of T cell response and anti-tumor immunity. Therefore, lipid metabolism is an important regulator of TME and ferroptosis of tumor cells (201). It is reported that prostaglandin E2(PGE2) can change the condition of TME by inhibiting the functions of NK cells, cytotoxic T cells and conventional type 1 dendritic cells (cDC1), which can further affect lipid metabolism (202).

In addition, related studies have found that there may be an endogenous trigger of ferroptosis in tumor cells-cystine limitation (203). That is, the degradation of cysteine induced by cyst(e)inase treatment will lead to ferroptosis of tumor cells, which is due to the decrease of GSH and the increase of ROS (204). The metabolic activity of tumor can also affect the changes of TME, and immune escape can be achieved by inhibiting the function of T cells (205, 206). The results show that the metabolic response triggered by T cells can affect the terminal fate of tumor cells. Therefore, improving the metabolism related to ferroptosis in tumor is expected to improve the effect of tumor immunotherapy and new insights.

#### 3.2.2 The interaction mechanism and treatment strategy of the tumor immune microenvironment and ICD

TIME dynamically regulates the initiation and effector phases of ICD through a complex immunosuppressive network, acting as a key barrier to limit the anti-tumor immune response. In tumors with low immune infiltration (i.e., “cold tumors”) or immune exclusion, the immunosuppressive properties of TIME significantly impair the efficiency of ICD. This is manifested by insufficient infiltration of antigen-presenting cells (APCs), which results in the failure to effectively capture DAMPs (e.g., ATP, HMGB1) released by dying tumor cells, thereby inhibiting cross-presentation of antigens and activation of cytotoxic T lymphocytes (CTLs) (82). In addition, co-inhibitory receptors highly expressed in TIME (e.g., CTLA-4, TIM-3) directly block APC recognition of “eat me” signals (e.g., calreticulin) by binding HMGB1 or phosphatidylserine (82). Metabolic reprogramming further exacerbates immunosuppression, such as the conversion of extracellular ATP to adenosine mediated by the CD39/CD73 axis, which not only inhibits CTL function but also promotes T cell exhaustion by activating the adenosine A2A receptor (ADORA2A) (207).

Despite the multiple suppressive factors in TIME, ICD can be activated by the release of DAMPs to activate an adaptive immune response. The specific mechanisms include: DAMPs can activate dendritic cells (DCs) to remodel the immune response network and coordinate multidimensional antitumor immune effects (208). When DAMPs, such as HMGB1 and ATP, bind to pattern recognition receptors (PRRs) on the surface of DCs (e.g., TLR and NLRP3), they trigger DC maturation, enhance antigen processing and presentation capabilities (86), and promote the secretion of proinflammatory factors such as IL-12 and TNF-α, as well as CCR7-mediated lymph node homing. Activated DCs directly activate CD8+ T cells through the MHC-costimulatory signaling axis, inducing their differentiation into cytotoxic T lymphocytes (CTLs) (10), and regulate the polarization of CD4+ T cell subsets (e.g., Th1/Th17/Treg) to adapt to the demands of the immune microenvironment (154). In addition, DCs drive B cell differentiation into plasma cells through the secretion of BAFF/APRIL and cell contact-dependent signals, promoting the production of high-affinity antibodies (166, 167). They also release CXCL8/GM-CSF to recruit neutrophils and enhance their phagocytic and NET formation abilities (181). Exosome-mediated antigen information exchange and IFN-γ signal transduction between DCs and macrophages further amplify the proinflammatory and antigen-presenting functions of M1-type macrophages (193). This cascade reaction initiated by the DAMPs-DC axis not only strengthens the synergistic effects of innate and adaptive immunity but also provides a theoretical framework for DC-targeted vaccine design or combined immune checkpoint blockade therapy (209). For example, CRT and other DAMPs can be used to enhance the efficacy of DC vaccines, or IL-10 signaling can be blocked to reverse the immunosuppressive microenvironment, thereby optimizing the anti-tumor immune response (210).

In addition, ATP drives DC maturation and recruits CTLs to the tumor site by binding to the purine receptor P2RX7; and HMGB1 activates Toll-like receptor 4 (TLR4) to enhance pro-inflammatory signaling to accelerate the ICD process (207). However, the inhibitory factors in TIME (such as IL-10 and TGF-β1) and the functional defects of immature DCs lead to a 30%-50% decrease in antigen cross-presentation efficiency (211). To break through this bottleneck, a combined intervention strategy has emerged: Shikonin (SHK) and curcumin (CUR) form a self-delivering nanosystem (chitosan-polyethylene glycol nanoparticles, CS-PEG NPs) that synergistically induces endoplasmic reticulum stress and Ca^2+^ homeostasis imbalance, which significantly increases the release of DAMPs, thereby increasing DC maturation and significantly increasing the infiltration density of CTLs in the core region of the tumor (from 5% to 20%) (211), Thus reversing the immunosuppression mediated by regulatory T cells (Tregs) and myeloid-derived suppressor cells (MDSCs). DMAPs remodel the tumor immune microenvironment by inducing immunogenic cell death. Under stress, tumor cells release DAMP molecules (CRT, HMGB1, ATP, and type I IFN). CRT acts as an “eat-me” signal to promote antigen-presenting cells to phagocytose tumor antigens; HMGB1 enhances dendritic cell maturation through TLR4; ATP activates the NLRP3 inflammasome to drive CD8+ T cell activation; type I IFN enhances NK cell killing function through the cGAS-STING pathway while inhibiting Treg activity, thereby reversing immune suppression and restoring the body’s antitumor capacity. These combined effects indicate that DMAPs have significant antitumor potential in TIME remodeling (212, 213).

**Figure 1:**
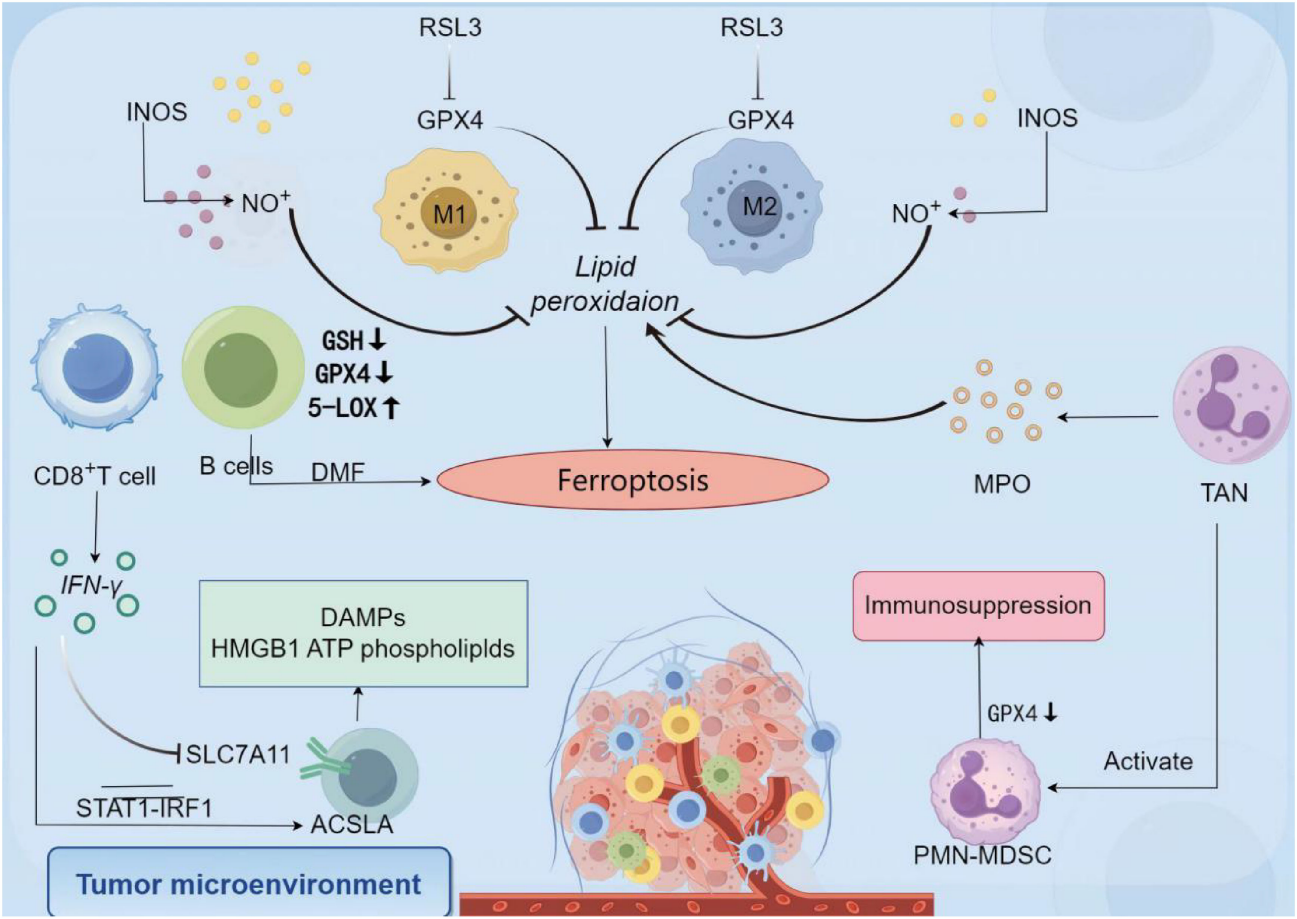
Schematic diagram of cross-regulation of ferroptosis and immunogenic cell death in tumor microenvironment. (In the tumor microenvironment, ferroptosis and immunogenic death are cross-regulated through complex mechanisms. In the core pathway of ferroptosis, RSL3 can regulate the GPX4 levels of M1 and M2 macrophages, causing lipid peroxidation and thus promoting ferroptosis. The specific mechanism is as follows: M1 macrophages highly express INOS, which catalyzes the production of a large amount of NO, which promotes lipid peroxidation—a core process of ferroptosis—thereby promoting ferroptosis of tumor cells; M2 macrophages have low expression of INOS and produce little NO, which makes it difficult to effectively induce lipid peroxidation and inhibit ferroptosis. Meanwhile, DMF further promotes lipid peroxidation by reducing intracellular GSH and GPX4 levels and upregulating 5-LOX, which strengthens the ferroptosis process. In terms of immunogenic death, tumor cells release DAMPs such as HMGB1 and ATP when undergoing ferroptosis, and these molecules can activate the immune response. For example, IFN-γ secreted by CD8+ T cells regulates SLC7A11 and ACSL4 through the STAT1-IRF1 pathway, which not only affects ferroptosis-related metabolism but also promotes immune activation. The cross-regulatory network between the two is manifested in many ways. On the one hand, immune cells are involved in the regulation of ferroptosis. M1 and M2 macrophages affect lipid peroxidation through GPX4, thereby regulating the occurrence of ferroptosis. Neutrophil-derived substances such as MPO are also involved in the ferroptosis process. On the other hand, ferroptosis affects the immune microenvironment. Ferroptosis-related signals such as GPX4 downregulation can activate immunosuppressive cells such as PMN-MDSC, leading to immunosuppression. The release of DAMPs, on the other hand, activates the immune response, forming a two-way regulation of “ferroptosis-immune activation-microenvironment remodeling” that ultimately affects tumor development). (Created by Figdraw).

The dynamic interaction between TIME and ICDs not only serves as a barrier to immunotherapy, but also provides potential targets for the development of new combination therapies. When ICDs are used in combination with immune checkpoint inhibitors (ICIs), the neoantigens they release, together with DAMPs, provide a dual “antigen-adjuvant” signal, while anti-PD-1/CTLA-4 antibodies form a triple “antigen-adjuvant-unblocking of inhibition” effect by relieving the inhibitory state of T cells (85). For example, irreversible electroporation (IRE) significantly alleviates the immunosuppressive microenvironment of pancreatic cancer by disrupting the integrity of tumor cell membranes, increasing the release of DAMPs such as HMGB1, and downregulating PD-L1 expression (108). Paclitaxel nanocarriers convert “cold tumors” into highly immunoinfiltrated “hot tumors” by inhibiting STAT3 phosphorylation and polarizing M2 tumor-associated macrophages (TAMs) to the proinflammatory M1 phenotype, increasing the infiltration of CTLs from 15% to 45% (108). The above studies have shown that targeting key TIME nodes such as DAMP release, metabolic reprogramming, and immune checkpoints can effectively enhance the efficacy of ICD. Future research needs to further elucidate the impact of TIME heterogeneity on ICD and develop combined delivery systems with temporal and spatial control characteristics to overcome immunosuppressive networks. As shown in **Figure 1**.

#### 3.2.3 The tumor microenvironment bidirectionally regulates ferroptosis and immunogenic cell death through metabolic stress, hypoxia, and cytokine networks

##### 3.2.3.1 Threshold effect of metabolic stress

Nutritional deprivation in the TME synergistically regulates ferroptosis and (ICD through multidimensional mechanisms. Cysteine competitive depletion inhibits GSH synthesis, leading to inactivation of GPX4 and driving lipid peroxidation-dependent ferroptosis; this process is exacerbated under glucose deprivation conditions due to AMPK-mediated fatty acid oxidation (FAO) pathway activation, forming a positive feedback loop of lipid peroxidation substrate accumulation (Kshattry et al., 2019; Liu et al., 2021) (202, 204). Concurrently, nutritional deprivation triggers endoplasmic reticulum stress (ERS), promoting the surface exposure of calretin (CRT) via the unfolded protein response (UPR), thereby enhancing the immunogenicity of tumor cell ICD (Garg et al., 2012) (208). However, when nutritional deprivation exceeds a critical threshold, ATP synthesis impairment leads to insufficient release of DAMPs, inhibiting antigen cross-presentation by dendritic cells (DCs), and ultimately weakening the immune activation efficacy of ICD (Krysko et al., 2012) (214). This dynamic equilibrium suggests that the intensity of metabolic stress is a key determinant of the direction of tumor immune editing.

##### 3.2.3.2 Spatiotemporal regulation of hypoxia signals

Hypoxia differentially regulates ferroptosis and ICD through the HIF signaling pathway. At the level of ferroptosis, hypoxia inhibits lipid peroxidation through a dual mechanism: 1) HIF-1α/2α upregulates PLIN2, a lipid droplet-associated protein, forming a physical barrier to isolate peroxidation reaction substrates (Zou et al., 2020) (215); 2) IDH1/2-dependent reverse TCA cycle activates the NADPH-GSH antioxidant system, enhancing reducing power reserves (He et al., 2024) (200). The regulation of ICD is time-dependent: acute hypoxia promotes HMGB1/ATP release through mitochondrial ROS bursts, enhancing DC antigen presentation function (Semenza et al., 2012) (216); chronic hypoxia significantly reduces CD8+ T cell infiltration through HIF-1α-mediated immunosuppressive metabolic reprogramming, blocking the immune effects of ICD (Noman et al., 2014) (217). This spatiotemporal heterogeneity reveals the dynamic regulatory nature of hypoxia in tumor immune escape.

##### 3.2.3.3 Immunometabolic regulation of cytokine networks

The cytokine profile in the TME precisely regulates the interaction between ferroptosis and ICD by shaping the pro-inflammatory/anti-inflammatory balance. Pro-inflammatory factors such as IFN-γ weaken GSH synthesis by downregulating the cystine transporter SLC7A11, thereby enhancing ferroptosis sensitivity (Wang et al., 2020) (10); TNF-α releases DAMPs such as HMGB1 through necroptotic apoptosis, forming synergistic immune activation with ICD (Vandenabeele et al., 2022) (209). In contrast, the immune-suppressive factor TGF-β enhances antioxidant defense through the Smad3/GPX4 pathway (Tang et al., 2021) (19), while IL-10 blocks immune surveillance by inhibiting DC maturation (Vegran et al., 2011) (210). Notably, the IL-6/JAK2/STAT3 pathway exhibits bidirectional regulation: it promotes the accumulation of PUFAs by activating fatty acid synthase (FASN), thereby increasing the risk of ferroptosis, while simultaneously inhibiting CRT exposure to weaken the immunogenicity of ICD (Johnson et al., 2020) (201). This multi-layered cytokine interaction network provides a theoretical basis for combined therapeutic strategies targeting the TME.

#### 3.2.4 Regulatory role of immune cells

##### 3.2.4.1 Neutrophil granulocyte

Tumor-associated neutrophils (TANs) regulate ferroptosis in tumor cells through multiple mechanisms. Their direct mechanisms of action include iron overload mediated by NETosis and oxidative damage by myeloperoxidase (MPO). TANs promote lipid peroxidation driven by the Fenton reaction by forming neutrophil extracellular traps (NETs), releasing DNA-histone complexes and capturing free iron in the microenvironment, thereby increasing local iron concentration by 2–3 times. In addition, MPO secreted by TANs can catalyze the production of hypochlorous acid (HOCl) from H_2_O_2_ and Cl⁻, which directly oxidizes tumor cell membrane lipids, resulting in a 4-fold increase in lipid peroxidation and accelerating the ferroptosis process. In glioblastoma (GBM), TANs deliver MPO to tumor cells through cell membrane fusion or exosomes, stimulating a significant increase in lipid peroxidation markers such as MDA. This process is driven by DAMPs released by tumor necrosis: for example, HMGB1 recruits neutrophils to the necrotic area by activating the TLR4/NF-κB pathway, forming a positive feedback loop of “necrosis-DAMP-TAN infiltration” that further amplifies the ferroptosis effect (218, 219).

In addition to direct oxidative damage, TAN also indirectly affects the ferroptosis process by regulating iron metabolism. Under neuropathological conditions, impaired activity of peroxisome proliferator-activated receptor γ (PPARγ) can inhibit lactoferrin (Ltf) transcription, leading to increased free iron concentrations and increased lipid peroxidation levels in neurons, thereby exacerbating ferroptosis. This mechanism suggests that TAN may regulate tumor cell iron homeostasis by secreting iron-binding proteins such as Ltf. However, in a high-sugar microenvironment (such as a diabetes-related tumor), TAN Ltf secretion is inhibited by the ROS-JNK pathway, and its specific regulatory network still needs to be further analyzed (219). It is worth noting that the role of TAN is significantly tissue- and microenvironment-specific: for example, in breast cancer, TAN upregulates the expression of the ferroptosis-sensitive gene ACSL4 through CXCR2 signaling, which significantly increases the response rate of tumor cells to ferroptosis inducers (220).

In summary, TAN is involved in the ferroptosis molecular network through multiple pathways such as NETosis, MPO delivery, and iron metabolism regulation. Its function is dynamically regulated by the concentration of DAMPs, metabolic status, and cell-cell interactions. Future research needs to reveal the precise mechanism of TAN heterogeneity and its spatiotemporal interaction with ferroptosis, in order to provide theoretical support for novel anti-tumor strategies targeting the TAN-ferroptosis axis.

##### 3.2.4.2 T cell

In the tumor microenvironment, T cells regulate tumor cell ferroptosis through multidimensional molecular mechanisms. CD8+ T cells are the core effector cells that drive ferroptosis: after immune checkpoint inhibitors (such as anti-PD-L1 and anti-CTLA4) activate CD8+ T cells, IFN-γ they secrete binds to the tumor cell membrane receptor IFNGR1/2, activating the JAK-STAT1 signaling pathway. Phosphorylated STAT1 enters the nucleus and directly inhibits the cystine/glutamate antiporter system Xc=⁻ (composed of SLC7A11 and SLC3A2), resulting in a 60%-80% reduction in GSH synthesis and a 3-5-fold increase in lipid peroxidation levels due to impaired GPX4 activity (219, 221). At the same time, STAT1 forms a complex with interferon regulatory factor 1 (IRF1), upregulates ACSL4, and promotes the integration of polyunsaturated fatty acids (such as arachidonic acid) into the phospholipids of tumor cell membranes, significantly increasing lipid peroxidation sensitivity (156, 219). In addition, the granule enzyme B released by CD8+ T cells inactivates GPX4 by cleaving its C-terminal domain and degrades ferroptosis suppressor protein 1 (FSP1), resulting in a collapse of the tumor cell’s antioxidant defense system (222).

In contrast, regulatory T cells (Treg) inhibit the ferroptosis process through antagonistic mechanisms. Treg highly express GPX4 to maintain their own antioxidant capacity: GPX4 deficiency leads to increased accumulation of intracellular lipid peroxides and ferroptosis, thereby relieving immunosuppression of effector T cells (219). In addition, Treg limit the synthesis of GSH in effector T cells and tumor cells by competing for cystine and glutamine in the tumor microenvironment. For example, in a melanoma model, Treg infiltration significantly reduced tumor cell SLC7A11 expression, leading to resistance to ferroptosis (219).

The metabolic status of T cells and their sensitivity to ferroptosis are characterized by bidirectional regulation. Tumor-infiltrating CD8+ T cells mediate the uptake of oxidized lipids (such as oxysterols) through CD36, leading to increased intracellular lipid peroxide accumulation and inducing autophagic ferroptosis, which significantly reduces their antitumor activity. Inhibition of CD36 or overexpression of GPX4 can significantly increase the survival rate of CD8+ T cells (122, 222). It is noteworthy that CD8+ T cells are significantly more sensitive to GPX4 inhibition than tumor cells: ACSL4 knockdown significantly reduces ferroptotic cell death, while FSP1 overexpression provides protection through the CoQ10 regeneration pathway (156).

Based on the above mechanism, combined therapeutic strategies targeting the T cell-ferroptosis axis have shown clinical potential: radiotherapy synergizes with immunity, and radiotherapy-induced ATM activation synergistically inhibits SLC7A11 expression with IFN-γ released by CD8+ T cells, significantly increasing lipid peroxidation levels and thus significantly enhancing the therapeutic effect of ferroptosis (223); immune checkpoint inhibitors combined with ferroptosis inducers, anti-PD-1 combined with ACSL4 agonists (such as RSL3), upregulate ACSL4 through the STAT1-IRF1 pathway, making tumor cells more sensitive to T cell-mediated ferroptosis (219); photodynamic therapy (PDT) and immune memory, hemoglobin iron combined with PDT activate CD8+ T cells to release IFN-γ to induce ferroptosis, while the nano photosensitizer MAR releases tumor-associated antigens (TAAs) through ferroptosis, which increases the maturation rate of dendritic cells (DCs) and forms long-lasting anti-tumor immune memory (222, 224).

However, about 30% of tumor cells escape ferroptosis through SLC7A11 overexpression, leading to the failure of immunotherapy. Targeting the ferroptosis resistance pathway (e.g., in combination with the FSP1 inhibitor iFSP1) can reverse drug resistance and significantly improve the treatment response rate (219, 222). Future research should focus on the dynamic interaction mechanism between T cell metabolic reprogramming and ferroptosis in order to develop more effective combination treatment strategies.

##### 3.2.4.3 Macrophage

Macrophages are directly involved in the process of ferroptosis by regulating iron homeostasis. When red blood cells are abnormally increased or damaged, the macrophage surface receptor natural resistance-associated macrophage protein 1 (Nramp1) mediates red blood cell phagocytosis. After lysosomal digestion, heme oxygenase-1 (HO-1) breaks down heme to release free iron ions (Fe^2+^). Fe^2+^ produces ROS through the Fenton reaction, which increases lipid peroxidation levels by 2–3 times, thereby triggering ferroptosis in target cells (225–227). In addition, IL-6 regulates iron metabolism by activating the JAK-STAT3/BMP/SMAD pathway: after bone morphogenetic protein 6 (BMP6) binds to the membrane receptor, it induces phosphorylation of SMAD1/5/8 and upregulates hepcidin expression, resulting in the internalization and degradation of the iron transporter FPN, which exponentially increases the amount of intracellular iron accumulation, further exacerbating ferroptosis (228–230).

Macrophage polarization has a bidirectional regulatory effect on ferroptosis. M1 macrophages secrete TNF-α in response to LPS/IFN-γ stimulation, activate the NF-κB/MAPK pathway, upregulate NADPH oxidase 4 (NOX4), significantly increase ROS production, and inhibit the expression of the cystine/glutamate antiporter system Xc=⁻ (SLC7A11/SLC3A2) and GPX4, significantly enhancing ferroptosis sensitivity (221, 231). However, M1 macrophages highly express inducible nitric oxide synthase (iNOS), which catalyzes the production of nitric oxide (NO), and inhibit 15-LOX activity, thereby reducing lipid peroxidation levels and conferring ferroptosis resistance (232, 233). M2 macrophages activate the STAT6 pathway in response to IL-4/IL-13, upregulate ferritin to store free iron, and secrete TGF-β1. Smad3 signaling inhibits the function of the tumor cell system Xc=⁻, leading to a decrease in GSH synthesis and a doubling of the lipid peroxidation marker MDA level (221, 234, 235).

There is a significant synergistic effect between immune microenvironment remodeling and ferroptosis. Ferroptosis inducers (such as erastin) or nanoparticles (Fe_3_O_4_-SAS@PLT) can reverse the M2 polarization of tumor-associated macrophages (TAMs) and promote their conversion to the M1 phenotype, resulting in a significant increase in TNF-α and IL-12 secretion, respectively, and a significant improvement in the immunosuppressive microenvironment (222, 236). IL-6 regulates the polarization direction in a concentration-dependent manner: low concentrations activate STAT3 to induce M2 polarization, while high concentrations drive M1 polarization through STAT1, thereby regulating the ferroptosis process (237). In addition, extracellular vesicles released by M1 macrophages carry miR-140-5p, which targets and inhibits the stability of SLC7A11 mRNA in cardiomyocytes, inducing ferroptosis. Ferric citrate, on the other hand, antagonizes ferroptosis by activating the Nrf2 signal, promoting its nuclear translocation and binding to the antioxidant response element (ARE) in the GPX4 promoter region, which exponentially upregulates GPX4 expression (221).

Targeting macrophages in disease treatment strategies has made progress. In non-alcoholic steatohepatitis (NASH), Kupffer cells (KCs) in the liver are polarized to the M1 phenotype, which enhances lipid uptake, increases the level of free fatty acids in hepatocytes, and increases the accumulation of ROS, ultimately exacerbating ferroptosis (238). Inhibiting the TGF-β1/γ-glutamyl transpeptidase 1 (GGT1) axis in TAMs can significantly reduce iron-mediated hepatocyte death (227). In tumor therapy, engineered magnetic microspheres induce TAMs polarization to the M1 phenotype through the TLR4/NF-κB pathway, which reduces the expression of SLC7A11 in tumor cells and thus increases ferroptosis sensitivity (234). Nanoparticles that synergize with iron metabolism (such as chitosan-arabinogalactan/iron complexes CSAA/Fe@PPI) significantly enhance the ferroptosis effect by delivering exogenous Fe^3+^and inducing ferroptin autophagy, which increases the level of the LIP in cells and the lipid peroxidation marker 4-HNE (239).

**Figure 2:**
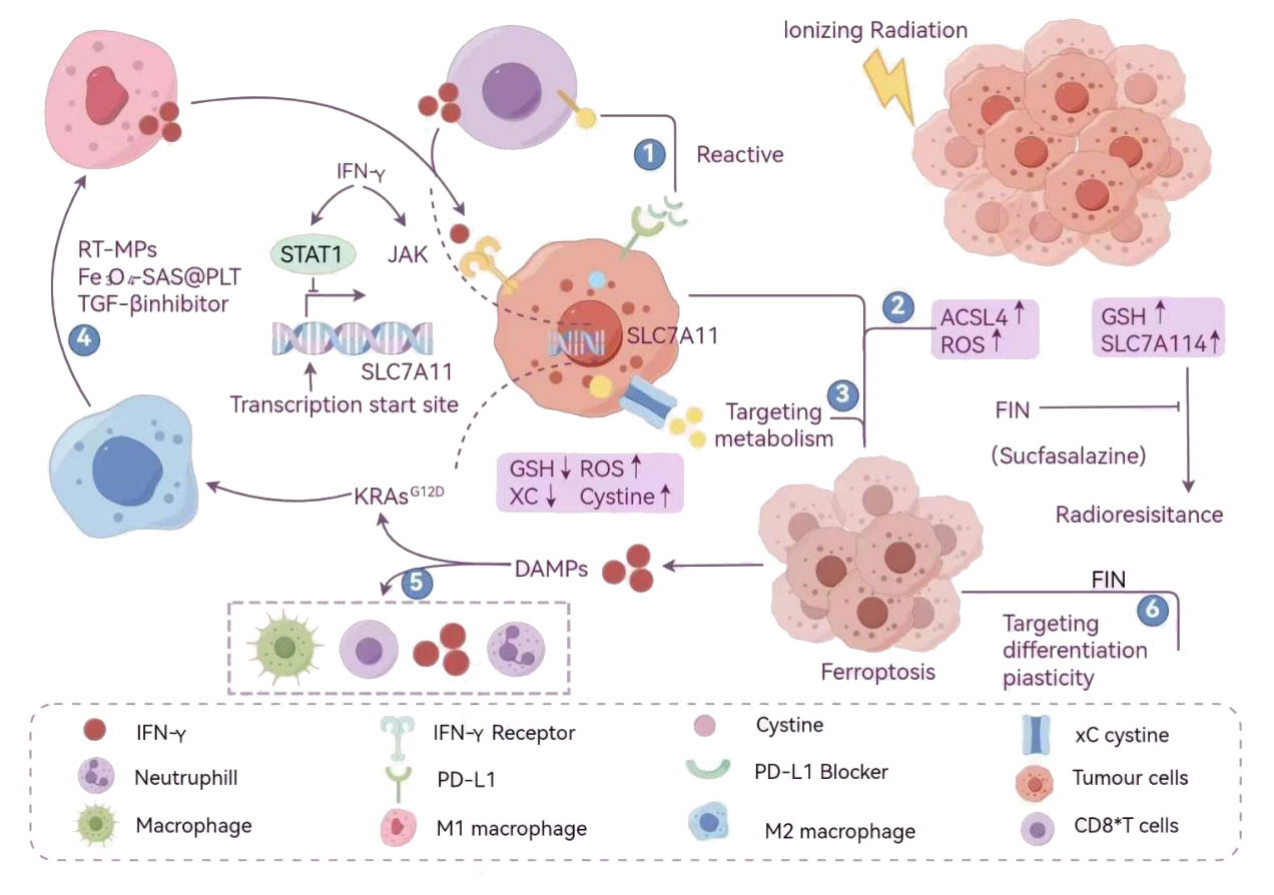
Comprehensive schematic diagram of cross-regulation of ferroptosis and immunogenic cell death. (Ionizing radiation activates the ferroptosis core pathway by triggering ROS bursts and lipid peroxidation: on the one hand, it upregulates ACSL4, which promotes the integration of PUFAs into cell membrane phospholipids and enhances oxidative sensitivity; on the other hand, it inhibits cystine/glutamate antiporter (system Xc⁻, composed of SLC7A11/SLC3A2), which leads to a decrease in GSH synthesis and loss of GPX4 activity, ultimately triggering ferroptosis. Radiation-induced tumor cell death releases DAMPs, such as ATP and HMGB1, which initiate antigen presentation and recruit CD8+ T cells to form an adaptive immune response. At the level of the immune microenvironment, ionizing radiation and the ferroptosis inducer PIN (sulfasalazine) can have a synergistic effect: IFN-γ inhibits SLC7A11 expression through the JAK-STAT1 signaling pathway, blocking the antioxidant defense of tumor cells; at the same time, the STAT1-IRF1 complex upregulates ACSL4, amplifying the lipid peroxidation effect and significantly increasing ferroptosis sensitivity. M1 macrophages secrete pro-inflammatory factors driven by IFN-γ to activate anti-tumor immunity; and PD-L1 blockers can relieve the inhibition of tumor cells on T cells and enhance the killing function of CD8+ T cells. Neutrophils release free iron and myeloperoxidase (MPO) through NETosis, directly oxidizing the tumor cell membrane and accelerating the process of ferroptosis. The comprehensive treatment strategy is a combination of RT-MPs, Fe_3_O_4_-SAS@PLT, TGF-β inhibitors, and other methods to regulate immune cells such as M2 macrophages and CD8+ T cells. By targeting metabolic plasticity and differentiation plasticity, a comprehensive anti-tumor mechanism of radiation combined with immune activation and ferroptosis induction is formed). (Created by Figdraw).

Although the ferroptosis axis targeting macrophages shows therapeutic potential, key issues still need to be addressed: developing nanoscale vectors with spatiotemporally specific regulation (such as pH or ROS-responsive), precisely regulating iron release and macrophage polarization; elucidating the ferroptosis escape mechanism caused by SLC7A11 overexpression or GPX4 mutation; exploring the impact of macrophage heterogeneity (such as the TREM2^+^ subset) on ferroptosis regulation and optimizing combined immunotherapy strategies. As shown in **Figure 2**.

In the tumor microenvironment, the interaction between ferroptosis and immune-competent cell death (ICD) has opened up a new frontier in cancer immunotherapy. Ferroptosis induces tumor cell death through lipid peroxidation, accompanied by the release of immune-stimulatory signals (such as ATP and HMGB1), forming a synergistic effect with ICD. This significantly enhances tumor antigen presentation and activates anti-tumor immune responses. This mechanism not only reshapes the immunosuppressive state of the tumor microenvironment but also provides key targets for breaking immune tolerance and improving the efficacy of immunotherapy.

In preclinical studies, several combination therapy strategies have achieved breakthrough progress and demonstrated significant potential for translation into clinical applications. The combination of ursolic acid and sorafenib inhibits SLC7A11 and reduces GSH synthesis, while significantly increasing lipid peroxidation levels, inducing ferroptosis in cells, and reversing drug resistance in liver cancer cells, thereby exhibiting significant antitumor activity and providing a new therapeutic strategy for tumor treatment. Zero-valent iron nanoparticles (ZVI-NP) can target and enhance the degradation of nuclear factor-E2-related factor 2 (NRF2), inducing ferroptosis in lung cancer cells through the Fenton reaction (with MDA levels increasing exponentially), while polarizing M2-type tumor-associated macrophages (TAMs) to M1-type and reducing the infiltration of regulatory T cells (Tregs) by half. This approach inhibits angiogenesis and weakens the self-renewal capacity of cancer cells, demonstrating great potential as a next-generation cancer therapy that reduces side effects and enhances efficacy (240, 241). In addition, liposomes containing prostaglandin 1 (LLI) activated systemic immune responses by inhibiting neutrophil ferroptosis and inducing intracellular cell death (ICD) in tumor cells, resulting in a significant reduction in the volume of distant metastatic lesions.

However, ferroptosis still faces multiple challenges in clinical application. Tumor heterogeneity leads to significant differences in the sensitivity of different cancer types to ferroptosis: gastric cancer is resistant due to low ACSL4 expression levels, while liver cancer is more sensitive due to high SLC7A11 expression levels. Immune cells also face heterogeneity issues: tissue-resident memory T cells (TRMs) often exhibit time-dependent heterogeneity in TRM exhaustion in late-stage tumors due to persistent antigen and TCR stimulation; depending on the distance from the tumor site, TRMs tend to increase in number with proximity, and TRMs can promote or inhibit the progression of different tumor types, exhibiting spatial heterogeneity (242). The immunosuppressive properties of the tumor microenvironment (TME) further limit its efficacy: regulatory T cells (Tregs) suppress ferroptosis by consuming cysteine and secreting IL-10, while tumor cells escape through the Nrf2 pathway by upregulating GPX4 and FSP1. In addition, ferroptosis inducers (such as Erastin) have low bioavailability and may cause liver and kidney damage (significant elevation of transaminases), highlighting the urgent need for the development of precise delivery systems. Similarly, immune cells exhibit varying sensitivity to ferroptosis inducers and interact with tumor cells, posing challenges such as selecting the appropriate inducer type and optimal dose, and developing treatment regimens that evolve with the tumor cycle. Overcoming these challenges is essential for the successful application of ferroptosis and ICD in tumor therapy.

Ferroptosis can activate antigen-presenting cells by releasing DAMP-related molecules, induce immune responses, and trigger anti-tumor immune memory through ICD. However, ferroptic death can also impair the function of immune effector cells, thereby affecting the efficacy of immune responses. Inflammatory factors activated by ICD and ROS produced by stress can promote the development of ferroptic death in tumor cells, thereby achieving anti-tumor effects. Based on the complex regulatory mechanisms of the two, many new therapies have emerged. Numerous novel combined strategies have emerged. Copper-based nanoparticles (HCuSPE@TSL-tlyp-1) induce ferroptosis through the Fenton reaction and release TLR7/8 agonists to activate dendritic cells (DCs), resulting in a significant increase in IFN-γ secretion and an increase in the proportion of CD8+ T cell infiltration. Manganese-based layered double hydroxide (Mn-LDH) nanosheets enhance IFN-γ secretion by activating the STING pathway, synergistically promoting ferroptosis and immune response, and prolonging mouse survival. Dynamic treatment regimens such as pH-responsive nanocarriers (CSAA/Fe@PPI) can precisely increase local tumor iron concentration while avoiding systemic toxicity. Immunotherapy combined with ferroptosis inducers is also one of the novel treatment strategies. Studies have shown that Erastin can reverse the M2 polarization of tumor-associated macrophages (TAMs) and promote their conversion to the M1 phenotype, significantly increasing TNF-α secretion to remodel the immunosuppressive microenvironment (222); BNP@R + L + aPDL1 (benzoic acid ester-functionalized nanoparticles loaded with RSL-3 + laser therapy + anti-PD-L1 antibody) can promote IFN-γ secretion in tumor tissues, inhibit the expression of endogenous SLC3A2 and SLC7A11, leading to a significant accumulation of ROS and lipid peroxides in tumor tissues, thereby promoting ferroptosis in cancer cells and increasing CD8+ T cell infiltration and DC cell maturation to reduce tumor cell metastasis and promote their ICD-mediated death (243).

The current experimental direction and design can focus on solving the problems faced by combined treatment of ferroptosis and ICD. Combined treatment faces the challenge of biological toxicity: ferroptosis can kill normal cells, while ICD may trigger cytokine storms and autoimmune diseases in patients. Faced with the problem of biological toxicity, the experimental direction can be to develop a targeted delivery system based on the tumor microenvironment response. By using nanocarriers to transport drugs, drugs can be released when stimulated by the acidic tumor microenvironment, achieving stimulatory targeted delivery and verifying its targeting *in vivo* and *in vitro*. Tumor heterogeneity causes differences in treatment effects, which is also a challenge for combined treatment: different tumor types may have huge differences in sensitivity to ferroptosis and ICD. The experimental direction can be to find the best inducers for different types of tumors and achieve personalized treatment plans according to the actual condition of patients. Through the design of multi-omics analysis of the molecular differences between sensitive and resistant cells, specific target verification can be carried out, and combined therapy using different inducers can be used for *in vivo* and *in vitro* experiments to find “subtyping-targeted” personalized combination therapies suitable for different tumors. Future research and development efforts can focus on two main directions: analyzing the molecular networks regulating ferroptosis in immune cells (such as TAMs and Tregs) within the tumor microenvironment (TME); and conducting precision clinical trials based on biomarkers (such as ACSL4 and SLC7A11). Despite numerous challenges, the synergistic role of ferroptosis and immune cell death (ICD) has opened up new dimensions for tumor treatment, and its successful translation will depend on the close integration of in-depth exploration of mechanisms and technological innovation.

**Document search**

1. Literature sources: PubMed (biomedical literature database), Web of Science (SCIE, Science Citation Index Expanded), Embase (biomedical and pharmacological literature database), Chinese Biomedical Literature Database (CBM), China National Knowledge Infrastructure (CNKI); the search period focused on January 1, 2019 to March 15, 2025. The language was limited to Chinese and English literature to ensure data homogeneity.
2. Search method: In order to more comprehensively search for literature that meets the criteria, a cross-search was conducted using keywords. The Chinese search terms were: (ferroptosis) AND (immunogenic cell death OR immune cell OR immunotherapy). The English search terms were: ferroptosis; ((Immunogenic cell death OR ICD) OR immune cell OR T cell OR B cell OR (neutrophile granulocyte OR neutrophil) OR (macrophage OR macrophagocyte Or megalophage); Tumor microenvironment, etc. The search strategy used a combination of subject terms (such as MeSH terms) and free terms, and Boolean operators (AND/OR/NOT) were used to construct an accurate search formula. The search syntax was adjusted for the characteristics of different databases (such as truncation characters in PubMed and synonym expansion in CNKI).
3. The initial search yielded a total of 9,411 relevant literature citations. Two researchers independently performed a preliminary screening of the titles and abstracts of all retrieved documents, and then read the full text of the documents screened in the first round to conduct a second screening based on the inclusion criteria, while recording the reasons for rejection. Duplicate citations were removed: all retrieved citations were imported into NoteExpress software for duplicate checking, and 8,731 were removed after deduplication. 7,966 were excluded in the first round of screening, and 765 were retained in the first round of screening.

(Preliminary screening: According to the exclusion criteria and inclusion criteria, the titles and abstracts of the retrieved entries are initially read to eliminate ineligible literature such as conference papers, technical reports, evaluation studies, and diseases that do not match.)

Secondary screening: According to the exclusion and inclusion criteria, the full text of the obtained literature was carefully read, and 514 articles that did not meet the inclusion criteria were excluded, and finally 251 articles that met the inclusion criteria were obtained. Inclusion criteria: The research content focuses on the cross-regulatory mechanism of ferroptosis and immunogenic cell death in the tumor microenvironment. The types of research include: basic experiments (cell or animal models) to reveal the molecular pathways of their interaction; clinical studies (such as cohort studies, RCTs) to explore the efficacy of combination therapy (such as ferroptosis inducers + immunotherapy); and mechanistic studies to analyze the effects of ferroptosis on the immune microenvironment (such as T cell infiltration and immune checkpoint expression). The types of literature are original research papers (Articles), reviews, meta-analyses, clinical studies, mechanistic studies, etc., and conference abstracts, reviews, and case reports are excluded. Exclusion criteria: The research subjects are non-tumor diseases (such as neurodegenerative diseases); the research content is not related to the tumor microenvironment; the data is incomplete or there are significant methodological deficiencies (such as insufficient sample size and no statistical analysis).Then, using specific data collection forms, the researchers extracted relevant data from each study, including basic information about the article (first author, year of publication, journal name, and type of study); the experimental model of the research design (e.g., cellular animal model or patient), interventions (e.g., erastin-induced ferroptosis, anti-PD-1 antibody therapy); key regulators of the core mechanism [e.g., Glutathione peroxidase 4 (GPX4), SLC7A11, adenosine triphosphate (ATP) release), signal pathways (e.g., nuclear factor-κB (NF-κB), mitogen-activated protein kinase (MAPK)]; and the main findings of ferroptosis’s promotion/inhibition of immunogenic cell death, remodeling effect on the tumour microenvironment, and synergistic effect of combination therapy, as well as the limitations of the experimental model (e.g., lack of clinical verification) and unresolved scientific questions.

Meta-analysis results showed that ferroptosis promotes immunogenic cell death by releasing damage-associated molecular patterns (DAMPs) (e.g., ATP, HMGB1). GPX4 is a central node of ferroptosis and immune regulation, and its low expression is associated with immune activation.
